# Trends, inequalities, and 2030 projections of women's tracheal, bronchial, and lung cancer attributable to air pollution, radon, and tobacco exposure

**DOI:** 10.3389/fgwh.2026.1662451

**Published:** 2026-08-07

**Authors:** Songsong Wang, Congcong Zhao, Hanpeng Lai

**Affiliations:** 1 Department of Public Health Monitoring and Evaluation, Yantai Center for Disease Control and Prevention, Yantai, China; 2 Department of Respiratory and Critical Care Medicine, Yangzhou Hongquan Hospital, Yangzhou, China; 3 School of Public Health, Yangzhou University, Yangzhou, China

**Keywords:** air pollution, disability-adjusted life year (DALY), female, mortality, radon, regional inequality, tobacco, lung cancer

## Abstract

**Background:**

Tracheal, bronchial, and lung (TBL) cancer attributable to environmental and behavioral exposures remains a major global health concern. However, evidence on long-term trends, inequalities, and future burden among women remains limited. This study used Global Burden of Disease (GBD) 2023 data to address these gaps.

**Methods:**

We analyzed female TBL cancer data from the GBD 2023 (1990–2023) to assess mortality and disability-adjusted life year (DALY) patterns associated with five rountine inhalation-related risk factors. We further evaluated associations with the socio-demographic index (SDI) using smoothing and deviation analyses, quantified SDI-related inequalities through concentration indices, decomposed burden changes by demographic and epidemiological factors, and projected the burden to 2030.

**Results:**

In 2023, smoking remained the leading contributor to female TBL cancer mortality, followed by ambient particulate matter and secondhand smoke. Between 1990 and 2023, mortality attributable to household solid fuel declined most markedly, whereas mortality related to particulate matter increased slightly. Comparable trends were observed for DALYs. Marked cross-country and regional heterogeneity was observed by risk factors, with China standing out for particulate matter-related burden. Along socio-demographic gradients, burdens from radon and tobacco-related hazards rose with SDI alongside elevated concentration indices, whereas solid fuel burdens declined with increasingly negative concentration indices, and particulate matter displayed a rising burden but diminishing inequality. Population growth and aging drove increases in burdens from particulate matter, radon, and tobacco, whereas epidemiological change reduced solid fuel-related burden. By 2030, burdens attributable to particulate matter, radon, and secondhand smoke are projected to continue increasing, whereas the burden related to solid fuel is expected to decline. Although the number of smoking-related cases is projected to increase, the corresponding rate is expected to decrease.

**Conclusions:**

Our findings underscore the need to prioritize surveillance of inhalation-related hazards for female TBL cancer, especially in resource-limited and rapidly developing regions beyond traditionally industrialized settings.

## Introduction

Tracheal, bronchial, and lung (TBL) cancer remains a major international public health issue due to its sustained and severe threat to human life and well-being, while also placing an enormous burden on social security systems (1). The female population has historically assumed caregiving roles and contributed substantially to family health maintenance. Therefore, the onset of TBL cancer places immense pressure on both patients and their family members, including increased financial strain and caregiving responsibilities. TBL cancer has become the second leading cause of cancer-related mortality in females, with approximately 0.8 million new cases and 0.7 million deaths globally in 2023, resulting in 15.1 million disability-adjusted life years lost (DALYs) (2). In recent years, population aging has further intensified mortality from TBL cancer among females, and this trend is expected to continue in the future. According to Global Cancer Statistics 2022, incident cases among females are projected to increase by approximately 83.3% by 2050, while deaths are expected to rise by about 96.2% (3).

Routine inhalation-related exposures represent an important but less frequently quantified component of the global TBL cancer burden. These exposures mainly occur through inhalation of harmful substances commonly present in everyday living environments, including indoor smoke, tobacco-related contaminants, and household or ambient particulate matter (4, 5). Socially assigned gender roles, particularly in less developed countries, contribute to disparities in exposure to routine inhalation-related hazards, which consequently lead to significant gender differences in the TBL cancer burden (6). In 2023, solid fuel use and secondhand smoke, which disproportionately affect women, accounted for 4.7% and 6.6% of DALYs due to female TBL cancer globally, respectively, both exceeding the corresponding proportions in males (4.0% and 4.3%) (2). These patterns suggest that differences in exposure to harmful substances may be associated with variations in the burden of TBL cancer among females. This trend may partly reflect the delayed health effects of long-term exposure, highlighting the need for gender-sensitive prevention strategies. Nevertheless, an integrated assessment of routine inhalation-related exposures in relation to female TBL cancer burden remains limited within existing Global Burden of Disease-based analyses. Variations in economic development can affect both inhalation-related exposure patterns and the implementation of chronic disease control strategies (7). The pattern of female TBL cancer burden arising from inhalation-related risk factors in both affluent and resource-limited regions warrants further investigation.

To address these gaps, we utilized GBD 2023 data on five common inhalation-related hazards associated with female TBL cancer and examined trends in disease burden across socioeconomic levels over the past 34 years to inform location-specific mitigation strategies. We also explored potential driving factors underlying changes in mortality and DALYs and projected the future trajectory of the disease burden.

## Materials and methods

### Data sources

The data utilized for our research were sourced from the Global Burden of Disease 2023 (GBD 2023) database (https://vizhub.healthdata.org/gbd-results/). Covering the period from 1990 to 2023 and spanning 204 countries and territories, this extensive database provided estimates on the incidence, prevalence, mortality, and disability-adjusted life years (DALYs) for 375 diseases and injuries, alongside insights into 88 specific risk factors contributing to the disease burden (8). The data sources employed to evaluate the distribution of TBL cancer burden by sex, age, location, and calendar year included diverse outlets such as censuses, disease registries, epidemiologic research, health service utilization data, hazard surveillance systems, and air pollution monitors (8). In this study, data on deaths and DALYs of TBL cancer attributable to specific risk factors were extracted from the GBD 2023. Since GBD data are publicly accessible and de-identified, ethical approval and informed consent were not required. (Additional file 1 provides detailed steps in searching data.) The socio-demographic index (SDI), developed based on the total fertility rate among individuals younger than 25 years, mean educational attainment among those aged 15 years or older, and lag-distributed income per capita, was used to reflect socioeconomic development status. According to SDI values, countries and territories were categorized into five regions: low SDI, low-middle SDI, middle SDI, high-middle SDI, and high SDI. (Additional file 2 provides detailed SDI results.) Additionally, 204 countries and territories were grouped into 21 GBD regions according to socioeconomic similarities and geographical proximity (9). Due to limited records for some subpopulations under the age of 30, the starting age for our collection was set at 30 years, extending up to 95 and above.

### Disease burden

In general, the International Classification of Diseases, Tenth Edition (ICD-10) codes relevant to TBL cancer include C33, C34-C34.92, Z12.2, Z80.1-Z80.2, and Z85.1-Z85.20 (10). The mortality-to-incidence ratio (MIR) was modeled by employing linear mixed-effects models incorporating covariates of the Healthcare Access and Quality (HAQ) Indices and was smoothed by the spatial-temporal Gaussian process utilizing time, age, and location as hyperparameters. Estimated mortality was obtained by multiplying incidence by the fitted MIR and was subsequently incorporated into the Cause of Death Ensemble model (CODEm), together with observed deaths from vital registration systems and verbal autopsies. CODEm produced the final mortality estimates by leveraging all accessible data and covariates, crafting a versatile array of plausible individual models that best fit the observed data (11). The four common sequelae for cancers, i.e., diagnosis/first therapy, controlled, metastatic, and terminal phases, alongside their corresponding disability weights, were considered in our research. [Detailed sequelae are described in Additional file 3 (12)]. Years lived with disability (YLDs) were calculated by multiplying the prevalence of specific health states by their corresponding disability weights, whereas years of life lost (YLLs) were estimated by multiplying the number of deaths at each age by the difference between standard life expectancy and age at death, together constituting DALYs (13).

### Risk factors

In the GBD 2023 study, risk-outcome pairs that have been evaluated as meeting the convincing or probable evidence standards established by the World Cancer Research Fund (WCRF) were incorporated to compile relative risk data (14). In our current study, exposure to ambient particulate matter pollution, household air pollution from solid fuels, residential radon, smoking, and secondhand smoke were collectively defined as selected risk factors contributing to TBL cancer. Estimates of household air pollution from solid fuels, smoking, and secondhand smoke were primarily informed by large-scale population-based surveys. Estimates of ambient particulate matter pollution incorporated geographic information system (GIS) data, while estimates of residential radon exposure were derived from national indoor monitoring data and published literature, as integrated within the GBD risk assessment framework.

### Statistical analysis

Given our study's focus on individuals aged 30 and above, not the entire population, age-standardized mortality rates (ASMRs) and age-standardized DALY rates (ASDRs) were recalculated using the GBD standard population defined by the Institute for Health Metrics and Evaluation (IHME) as a reference (15). All re-estimated rates included 95% uncertainty intervals (UIs) derived from the empirical distribution of 100,000 Monte Carlo simulations, computed as the 2.5th and 97.5th percentiles of each computational step (16). Therefore, the resulting estimates are not directly comparable with the all-age estimates reported in the GBD Results Tool. (Further details on the recalculation and validation are provided in Additional file 4 and Additional file 5.).

For estimating the trends of ASMR and ASDR within a predetermined interval, average annual percentage changes (AAPCs) were calculated by using joinpoint regressions. The optimal points were identified by the changing slopes and were linked to a set of logarithmic linear models with a maximum of five joint points (11). The equation was defined as follows:

$$E(y|x)=e^{\beta_{0}+\beta_{1}x+\delta_{1}(x-\tau_{1})+\ldots +\delta_{k}(x-\tau_{k})+\ldots }$$

where *k* indicated number of turning points, *­τ_k_* indicated unknown turning points, *­β*_0_ indicated constant, ­*­β*_1_ indicated regression coefficient, and *δ­_k_* indicated regression coefficient of the *k*th piecewise function.

The correlations between age-standardized rates and SDIs were assessed using the Spearman rank test, and deviation analysis was performed through locally estimated scatterplot smoothing (LOESS) to model the ASR-SDI association, derive expected values, and calculate residuals (observed minus predicted) to identify countries with higher-than-expected burdens. The concentration indices were adopted to quantify the distributive inequality of burden across nations, calculated by integrating the area under the fitted Lorenz concentration curve, with the cumulative burden and the cumulative population distribution ranked by SDI. The decomposition method was applied to separate the contributions of population growth, ageing, and epidemiological changes to the shifts in disease burden (17).

The Bayesian Age-Period-Cohort (BAPC) model was adopted to project the disease burden from 2024 to 2030, employing integrated nested Laplace approximations (INLA) to estimate marginal posterior distributions and thus avoid the mixing and convergence issues associated with traditional Bayesian methods. The model is formulated within a Bayesian framework to capture age, period, and cohort effects, with the assumption that these effects evolve smoothly over time. Predictive uncertainty was quantified using 95% credible intervals, and the projected estimates should be interpreted as model-based forecasts rather than exact future observations (18).

All analyses were performed using R version 4.5.2 software (Institute for Statistics and Mathematics, Vienna, Austria). A two-sided *P* value < 0.05 was considered statistically significant.

## Results

### Spatiotemporal trends of burden by Air pollution, radon, and tobacco exposure

In 2023, the highest ASMR of female TBL cancer was observed for smoking [9.07 (8.22, 9.92) per 100,000], followed by ambient particulate matter [3.75 (3.22, 4.29)] and secondhand smoke [2.75 (2.50, 2.99)] (Table 1). From 1990 to 2023, ASMR from ambient particulate matter increased slightly [AAPC = 0.66 (0.62, 0.70)], while ASMRs for the other selected risk factors declined, most markedly for household solid fuel use [AAPC = −4.01 (−4.11, −3.90)] (Table 1). ASDR showed patterns of ranking and temporal variation similar to those observed for ASMR (Supplementary Table S1). In 2023, the regions with the highest ASMRs varied by risk factor, with East Asia highest for ambient particulate matter, Central Sub-Saharan Africa for household solid fuel use, Central Europe for residential radon, and High-income Asia Pacific for smoking (Table 1). The regional distribution of ASDR in 2023 largely mirrored ASMR, except that High-income North America, rather than High-income Asia Pacific, had the highest ASDR for smoking (Supplementary Table S1).

**Table 1:** ASMRs (per 10^5^) of female TBL cancer attributable to air pollution and tobacco exposure in 2023 with AAPCs from 1990 to 2023.

Location	Ambient particulate matter pollution		Household air pollution from solid fuels		Residential radon		Smoking		Secondhand smoke	
	ASMR in 2023	AAPC (1990–2023)	ASMR in 2023	AAPC (1990–2023)	ASMR in 2023	AAPC (1990–2023)	ASMR in 2023	AAPC (1990–2023)	ASMR in 2023	AAPC (1990–2023)
Total Females	3.753 (3.217, 4.286)	0.657 (0.616, 0.696)	1.020 (0.771, 1.270)	−4.006 (−4.108, −3.904)	1.175 (0.596, 1.756)	−0.211 (−0.237, −0.187)	9.072 (8.222, 9.918)	−0.855 (−0.904, −0.810)	2.746 (2.504, 2.990)	−0.759 (−0.871, −0.654)
SDI Region										
Low SDI	0.802 (0.661, 0.941)	3.032 (2.988, 3.073)	2.817 (2.378, 3.257)	0.822 (0.776, 0.868)	0.398 (0.199, 0.596)	1.539 (1.490, 1.582)	1.249 (1.014, 1.484)	0.848 (0.798, 0.895)	1.007 (0.879, 1.135)	1.246 (1.191, 1.295)
Low-middle SDI	1.500 (1.276, 1.722)	2.887 (2.838, 2.937)	3.028 (2.550, 3.509)	0.176 (0.128, 0.220)	0.597 (0.304, 0.889)	1.414 (1.356, 1.463)	2.311 (1.956, 2.662)	0.224 (0.163, 0.288)	1.519 (1.347, 1.690)	0.610 (0.545, 0.678)
Middle SDI	2.903 (2.497, 3.309)	3.219 (3.164, 3.271)	1.229 (0.904, 1.552)	−3.796 (−3.886, −3.717)	0.623 (0.320, 0.929)	0.429 (0.378, 0.477)	3.172 (2.749, 3.594)	−0.496 (−0.586, −0.425)	2.999 (2.714, 3.282)	0.476 (0.419, 0.528)
High-middle SDI	5.171 (4.401, 5.938)	2.039 (1.979, 2.100)	0.890 (0.470, 1.307)	−6.717 (−6.969, −6.359)	0.932 (0.465, 1.400)	−0.586 (−0.639, −0.532)	5.173 (4.129, 6.227)	−1.750 (−1.816, −1.698)	3.329 (2.969, 3.689)	−1.536 (−1.667, −1.408)
High SDI	4.326 (3.683, 4.963)	0.006 (−0.047, 0.050)*	0.250 (0.095, 0.406)	−6.418 (−6.679, −6.255)	1.658 (0.824, 2.497)	−0.143 (−0.173, −0.114)	15.109 (13.932, 16.292)	−0.524 (−0.559, −0.492)	3.087 (2.812, 3.365)	−0.854 (−0.900, −0.812)
GBD Region										
Central Asia	1.585 (1.393, 1.780)	−1.138 (−1.269, −1.006)	0.392 (0.306, 0.479)	−5.035 (−5.190, −4.870)	0.728 (0.353, 1.104)	−2.107 (−2.234, −1.965)	0.790 (0.628, 0.952)	−2.694 (−2.838, −2.509)	1.210 (1.114, 1.307)	−2.147 (−2.276, −2.016)
Central Europe	4.255 (3.652, 4.857)	0.235 (0.150, 0.316)	0.318 (0.138, 0.496)	−5.280 (−5.521, −5.088)	2.284 (1.058, 3.514)	1.400 (1.360, 1.436)	21.126 (19.712, 22.539)	1.480 (1.433, 1.518)	3.162 (2.899, 3.424)	0.251 (0.206, 0.290)
Eastern Europe	0.928 (0.792, 1.064)	−3.991 (−4.093, −3.875)	0.034 (0.019, 0.049)	−6.611 (−6.859, −6.351)	0.905 (0.461, 1.354)	−1.030 (−1.103, −0.939)	2.945 (2.530, 3.358)	0.316 (0.217, 0.409)	1.123 (1.029, 1.218)	−2.148 (−2.199, −2.085)
Australasia	1.125 (0.881, 1.368)	−0.650 (−1.223, −0.012)	0.017 (−0.010, 0.044)	−4.144 (−4.426, −3.946)	0.563 (0.212, 0.915)	−0.125 (−0.198, −0.053)	17.557 (15.795, 19.317)	−0.849 (−0.955, −0.768)	1.296 (1.181, 1.413)	−2.286 (−2.409, −2.199)
High-income Asia Pacific	2.294 (1.930, 2.662)	−0.126 (−0.243, 0.009)*	0.012 (−0.004, 0.028)	−5.515 (−5.622, −5.428)	0.586 (0.279, 0.892)	0.149 (0.072, 0.205)	6.727 (5.698, 7.755)	−1.071 (−1.149, −1.019)	1.738 (1.565, 1.912)	−2.554 (−2.648, −2.474)
High-income North America	1.486 (1.153, 1.816)	−4.414 (−4.538, −4.272)	0.010 (−0.005, 0.024)	−1.340 (−1.411, −1.269)	2.087 (0.972, 3.204)	−1.004 (−1.037, −0.970)	26.808 (24.681, 28.935)	−1.425 (−1.472, −1.382)	1.711 (1.564, 1.858)	−2.855 (−2.888, −2.824)
Southern Latin America	3.286 (2.838, 3.733)	1.267 (1.042, 1.494)	0.048 (0.003, 0.094)	−8.025 (−8.397, −7.829)	0.778 (0.280, 1.278)	1.008 (0.915, 1.090)	11.331 (9.980, 12.677)	0.741 (0.599, 0.843)	2.133 (1.958, 2.309)	−0.099 (−0.177, −0.014)
Western Europe	2.046 (1.681, 2.410)	−2.009 (−2.191, −1.829)	0.023 (−0.015, 0.061)	−2.486 (−2.592, −2.401)	2.277 (1.093, 3.451)	0.998 (0.953, 1.037)	20.705 (19.507, 21.906)	0.573 (0.519, 0.614)	1.745 (1.610, 1.879)	−0.635 (−0.673, −0.601)
Andean Latin America	2.905 (2.472, 3.337)	0.486 (0.312, 0.681)	0.603 (0.404, 0.804)	−4.202 (−4.519, −3.921)	0.761 (0.283, 1.236)	0.587 (0.473, 0.712)	1.839 (1.406, 2.270)	−0.252 (−0.379, −0.112)	0.831 (0.746, 0.916)	−0.788 (−0.917, −0.659)
Caribbean	1.791 (1.499, 2.079)	1.150 (0.937, 1.646)	0.852 (0.682, 1.021)	−2.473 (−2.553, −2.385)	0.569 (0.236, 0.905)	0.529 (0.453, 0.610)	11.534 (9.900, 13.175)	−0.092 (−0.254, 0.092)*	2.383 (2.134, 2.630)	0.085 (−0.021, 0.182)*
Central Latin America	1.744 (1.519, 1.970)	−1.491 (−1.583, −1.392)	0.371 (0.292, 0.451)	−4.577 (−4.666, −4.480)	0.722 (0.372, 1.075)	−1.158 (−1.220, −1.094)	2.638 (2.245, 3.032)	−2.866 (−2.919, −2.809)	0.926 (0.847, 1.006)	−2.762 (−2.819, −2.697)
Tropical Latin America	2.216 (1.904, 2.527)	0.925 (0.817, 1.053)	0.146 (0.074, 0.217)	−6.148 (−6.444, −5.952)	1.028 (0.447, 1.611)	1.007 (0.938, 1.062)	10.402 (9.297, 11.509)	0.084 (−0.032, 0.159)*	1.490 (1.348, 1.633)	−1.357 (−1.454, −1.291)
North Africa and Middle East	3.482 (3.027, 3.942)	2.227 (2.113, 2.329)	0.276 (0.221, 0.330)	−4.205 (−4.259, −4.148)	0.607 (0.319, 0.896)	0.902 (0.863, 0.936)	3.113 (2.563, 3.663)	0.607 (0.531, 0.672)	2.027 (1.827, 2.228)	0.866 (0.827, 0.909)
South Asia	1.635 (1.343, 1.928)	4.563 (4.471, 4.649)	1.990 (1.653, 2.328)	−0.116 (−0.188, −0.053)	0.467 (0.233, 0.701)	1.936 (1.861, 2.013)	1.441 (1.137, 1.744)	0.911 (0.836, 1.005)	1.167 (1.014, 1.321)	0.922 (0.848, 0.994)
East Asia	8.634 (7.234, 10.049)	2.836 (2.681, 2.976)	1.398 (0.770, 2.031)	−6.434 (−6.602, −6.308)	1.530 (0.723, 2.342)	−0.397 (−0.456, −0.344)	10.162 (7.701, 12.641)	−1.176 (−1.267, −1.113)	5.855 (5.174, 6.540)	−1.025 (−1.161, −0.887)
Oceania	0.960 (0.767, 1.155)	1.706 (1.507, 1.993)	11.825 (9.461, 14.171)	0.844 (0.730, 0.945)	1.278 (0.269, 2.282)	1.198 (1.074, 1.292)	10.850 (8.351, 13.358)	0.690 (0.584, 0.776)	6.162 (5.185, 7.140)	1.335 (1.239, 1.421)
Southeast Asia	3.023 (2.576, 3.471)	2.136 (2.005, 2.324)	1.802 (1.433, 2.171)	−3.085 (−3.182, −2.985)	0.453 (0.232, 0.674)	0.709 (0.647, 0.782)	3.124 (2.597, 3.647)	−1.422 (−1.516, −1.320)	3.289 (2.943, 3.636)	0.263 (0.167, 0.374)
Central Sub-Saharan Africa	0.849 (0.682, 1.016)	2.387 (2.287, 2.480)	3.896 (3.051, 4.733)	1.214 (1.114, 1.303)	0.466 (0.108, 0.824)	1.546 (1.443, 1.635)	0.482 (0.334, 0.630)	1.219 (1.138, 1.313)	0.763 (0.624, 0.903)	1.417 (1.318, 1.508)
Eastern Sub-Saharan Africa	0.251 (0.212, 0.289)	2.591 (2.491, 2.699)	2.198 (1.877, 2.517)	1.079 (1.020, 1.134)	0.243 (0.119, 0.366)	1.459 (1.385, 1.523)	0.449 (0.356, 0.542)	0.916 (0.839, 1.018)	0.330 (0.290, 0.370)	0.452 (0.406, 0.497)
Southern Sub-Saharan Africa	2.368 (2.014, 2.720)	0.753 (0.625, 0.880)	0.884 (0.709, 1.058)	−2.516 (−2.622, −2.405)	1.003 (0.407, 1.589)	0.844 (0.722, 0.953)	6.656 (5.492, 7.809)	−0.344 (−0.512, −0.207)	2.429 (2.168, 2.695)	−0.260 (−0.397, −0.131)
Western Sub-Saharan Africa	0.848 (0.689, 1.008)	2.307 (2.164, 2.443)	1.813 (1.525, 2.099)	−0.174 (−0.254, −0.107)	0.332 (0.169, 0.494)	0.895 (0.829, 0.962)	0.265 (0.183, 0.347)	−2.144 (−2.227, −2.071)	0.329 (0.288, 0.369)	0.383 (0.312, 0.451)

AAPC, average annual percentage changes; ASMR, age-standardized mortality rate; TBL, tracheal, bronchus, and lung.

* The AAPC was NOT statistically significant since its *P* value > 0.05.

**Figure 1:**
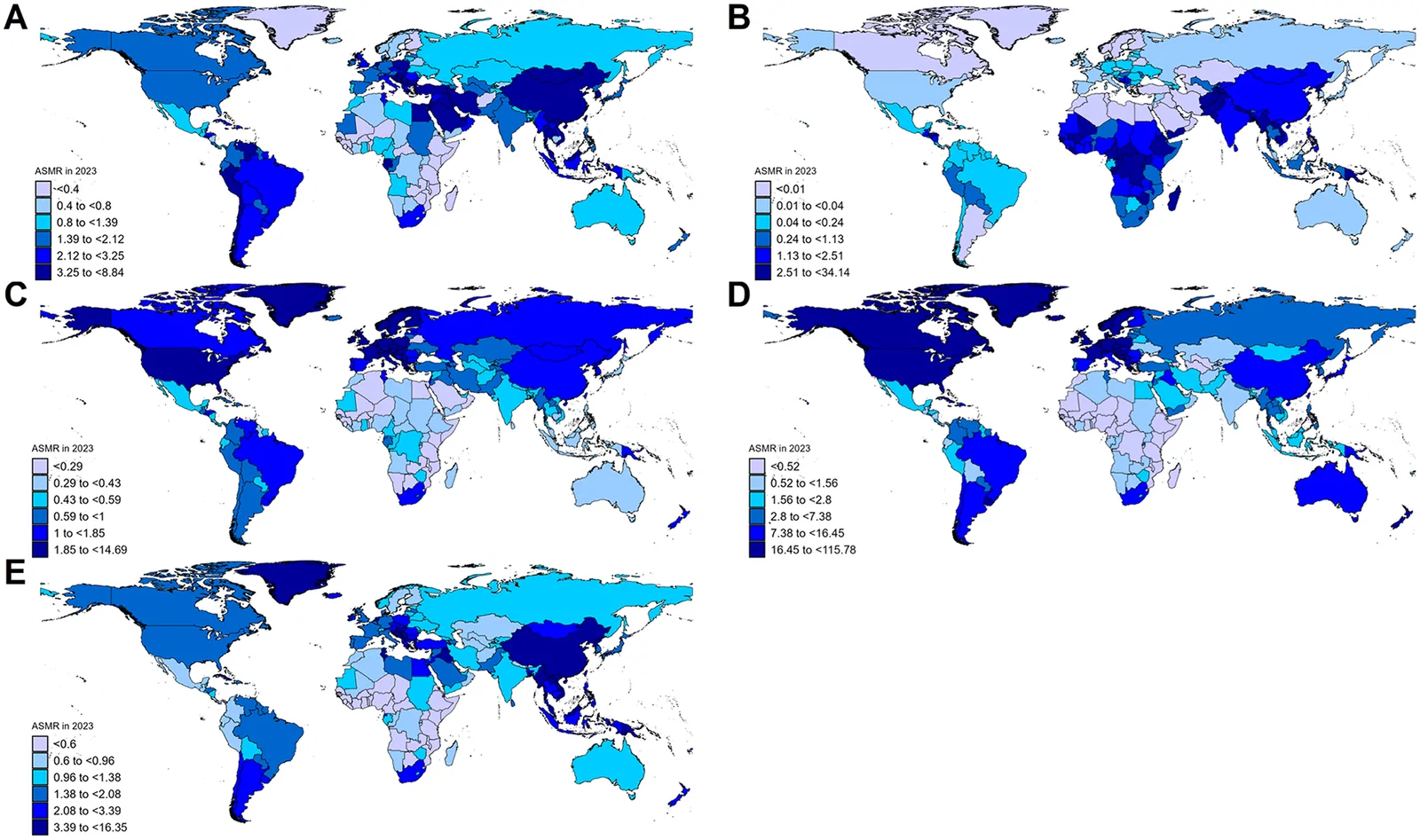
ASMR of female TBL cancer attributable to ambient particulate matter pollution **(A)**, household air pollution from solid fuels **(B)**, residential radon **(C)**, smoking **(D)**, secondhand smoke **(E)** in 2023, by countries.

In 2023, ambient particulate matter-related female TBL cancer had the highest ASMR in China, Central European nations, and the Middle East, all in the top sextile (Figure 1A). ASMRs attributable to household solid fuel and secondhand smoke were concentrated in the top sextile in Pacific Islands and low- and middle-income East Asia (Figures 1B,E). Central-Western Europe and the United States exhibited radon- and smoking-related ASMR in the highest sextile (Figures 1C,D). ASDR showed a similar country-level distribution in 2023 (Supplementary Figure S1).

**Figure 2:**
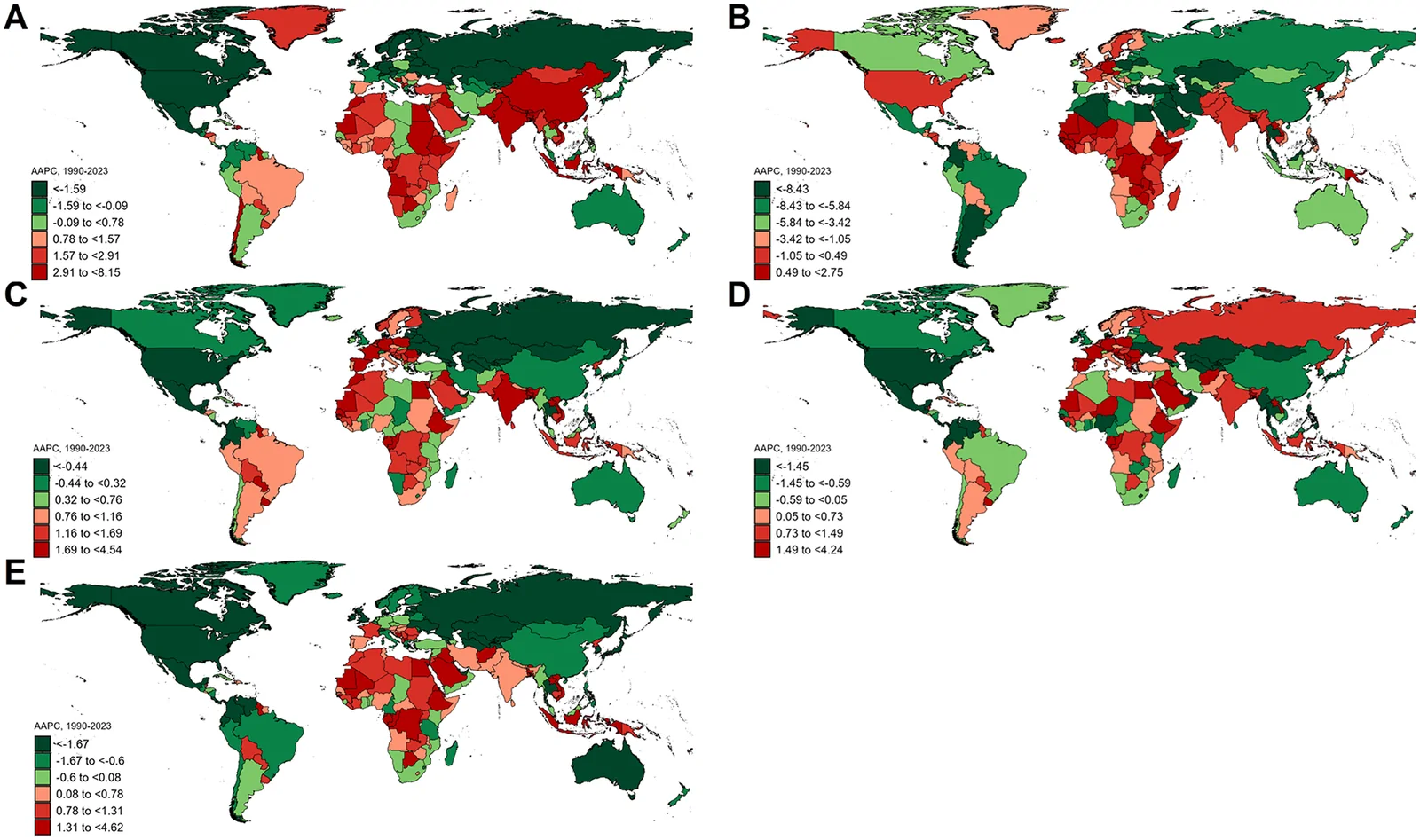
AAPC in ASMR of female TBL cancer attributable to ambient particulate matter pollution **(A)**, household air pollution from solid fuels **(B)**, residential radon **(C)**, smoking **(D)**, secondhand smoke **(E)** from 1990 to 2023, by countries.

From 1990 to 2023, the steepest declines in ASMR from ambient particulate matter, radon, and secondhand smoke were seen in North America, Eastern Europe, and Central Asia, with AAPCs in the lowest sextile (Figures 2A–E). The sharpest declines in ASMR from household solid fuels and smoking were observed in the Middle East and the United States, respectively (Figures 2B,D). Between 1990 and 2023, populous countries such as China, India, and Indonesia showed increases in ASMR from ambient particulate matter, while ASMR attributable to solid fuel use increased rapidly in sub-Saharan Africa (e.g., Ethiopia), with AAPCs falling in the highest sextile (Figures 2A,B). Smoking- and secondhand smoke-related ASMR increased in Central-Western Europe and Southeast Asia, respectively, with both also rising in Saudi Arabia (Figures 2D,E). India also experienced increases in radon-related ASMR (Figure 2C). Nation-level ASDR showed a consistent trend over 34 years (Supplementary Figure S2).

### Social gradients in burden by air pollution, radon, and tobacco exposure

**Figure 3:**
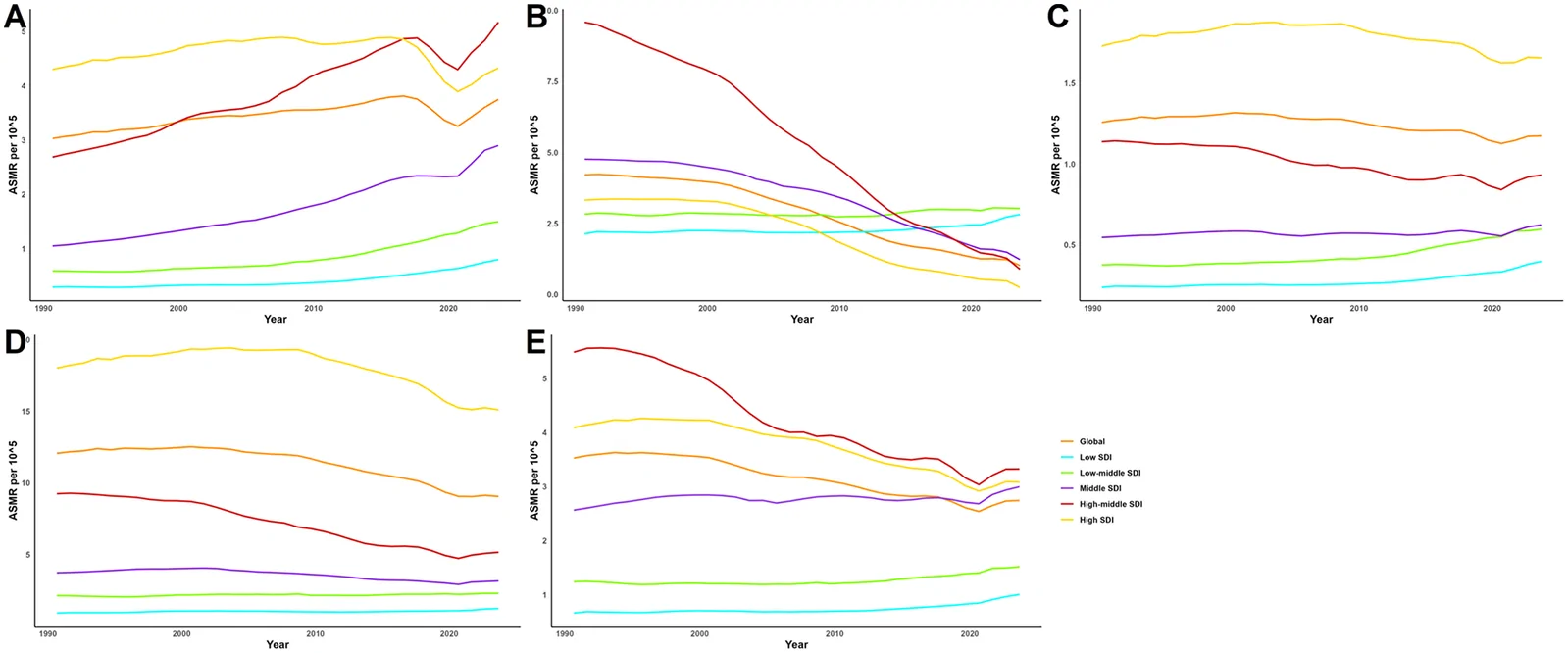
ASMR trend of female TBL cancer attributable to ambient particulate matter pollution **(A)**, household air pollution from solid fuels **(B)**, residential radon **(C)**, smoking **(D)**, secondhand smoke **(E)** from 1990 to 2023, by SDI regions.

In 2023, ASMRs and ASDRs of female TBL cancer from residential radon and smoking increased progressively with SDI, whereas burdens attributable to household solid fuel use and secondhand smoke declined to varying degrees in middle and higher SDI regions (Table 1, Supplementary Table S1). Over the past 34 years, ASMRs due to solid fuel use and smoking rose slowly in lower-SDI regions but declined markedly in middle- and higher-SDI regions, while radon- and secondhand smoke-related ASMRs continued to increase through middle-SDI regions before declining in high-middle and high-SDI regions (Tables 1; Figures 3B–E). Similar SDI-level patterns were observed for ASDRs in the past 34 years (Supplementary Tables S1; Figures S3B–S3E). Ambient particulate matter-related ASMR increased across all SDI regions, while ASDR declined in high-SDI regions [AAPC = −0.25 (−0.30, −0.21)] (Table 1, Supplementary Table S1; Figure 3A, Supplementary Figure S3A). Notably, in high-middle SDI areas, the burden from solid fuel fell from its peak, while the ambient particulate matter-related burden surged to the highest (Figures 3A,B, Supplementary Figure S3A, S3B).

**Figure 4:**
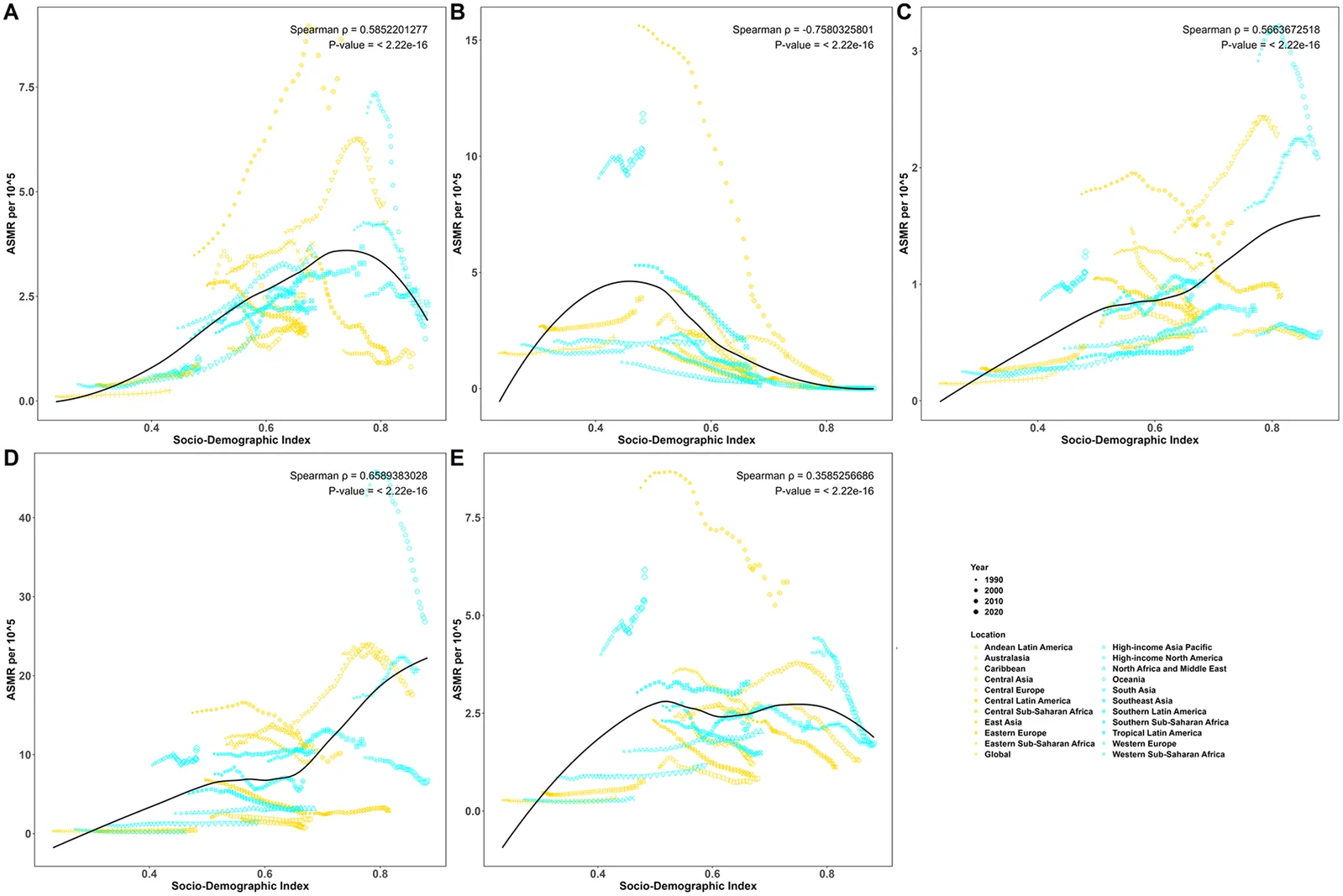
Correlations between SDIs and ASMRs of female TBL cancer attributable to ambient particulate matter pollution **(A)**, household air pollution from solid fuels **(B)**, residential radon **(C)**, smoking **(D)**, secondhand smoke **(E)** from 1990 to 2023, by GBD regions.

From 1990 to 2023, ASMRs of female TBL cancer attributable to ambient particulate matter, radon, smoking, and secondhand smoke across 21 GBD regions showed a nonlinear yet overall positive correlation with SDI, whereas ASMR from household solid fuel use was negatively associated with SDI (Spearman *ρ* = −0.76) (Figure 4). ASDR trends closely paralleled ASMR across SDI regions over 32 years (Supplementary Figures S4).

**Figure 5:**
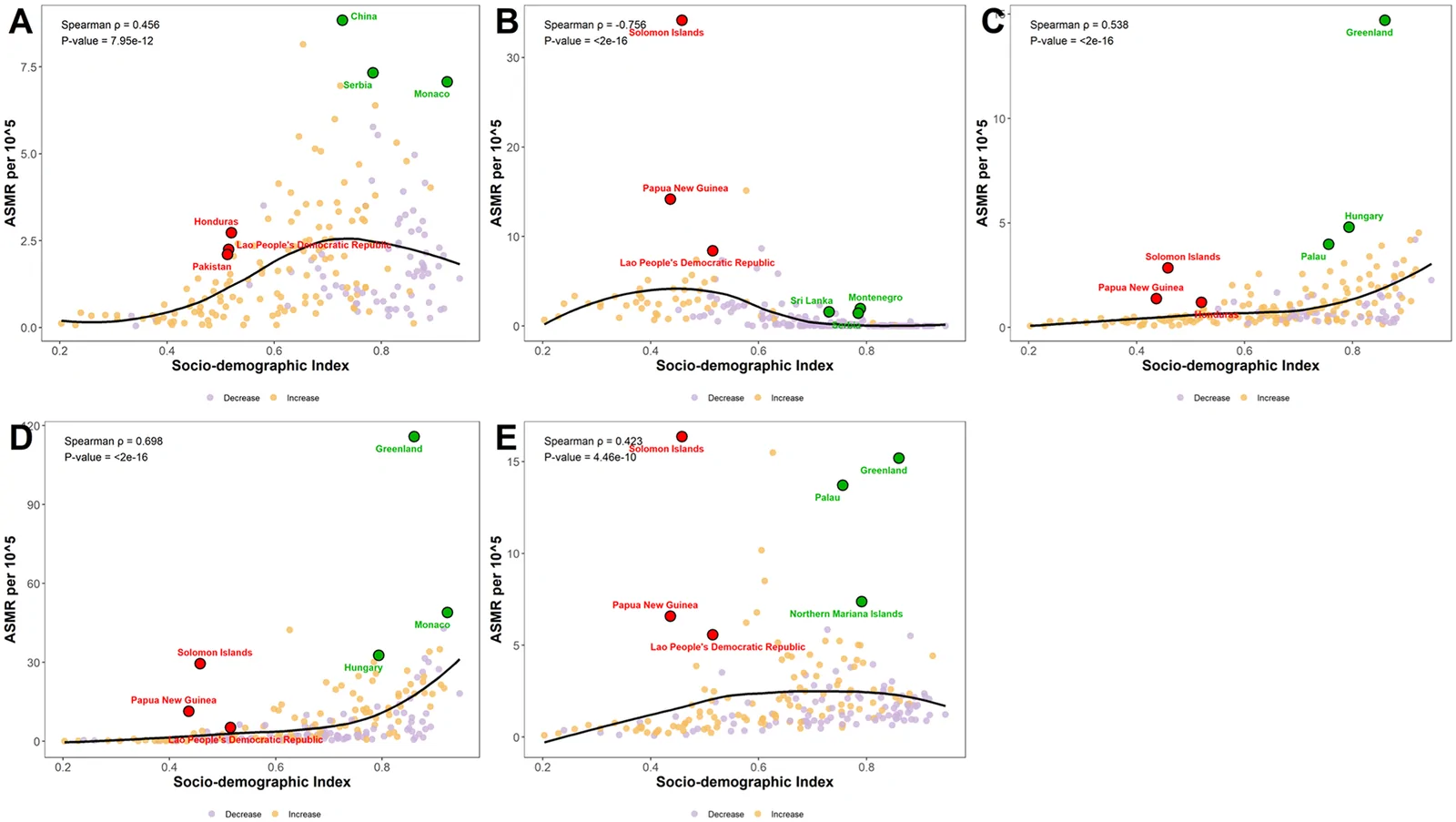
Correlations between SDIs and ASMRs of female TBL cancer attributable to ambient particulate matter pollution **(A)**, household air pollution from solid fuels **(B)**, residential radon **(C)**, smoking **(D)**, secondhand smoke **(E)** in 2023, by countries. A LOESS curve was fitted to estimate the expected ASMR by SDI, and deviation was calculated as the difference between observed and predicted values. The three countries with the largest positive deviations within the low-SDI (SDI < 0.5316) and high-SDI (SDI > 0.7188) groups were labeled. Dot colors indicated whether ASMR increased or decreased between 1990 and 2023.

In 2023, across 204 nations, ASMRs of female TBL cancer due to ambient particulate matter, radon, smoking, and secondhand smoke were significantly positively correlated with SDI, except for solid fuel use (Spearman *ρ* = −0.76) (Figure 5). Among nations with SDI > 0.72, the observed ASMR from ambient particulate matter exceeded the LOESS-predicted value most markedly in China, with the largest deviations for radon, smoking, and secondhand smoke found in Greenland (Figure 5). The Solomon Islands showed the greatest deviations from LOESS-predicted ASMRs for all selected risk factors except ambient particulate matter in countries with SDI < 0.53 (Figure 5). Similar SDI-related patterns appeared for ASDRs across nations in 2023 (Supplementary Figure S5).

**Figure 6:**
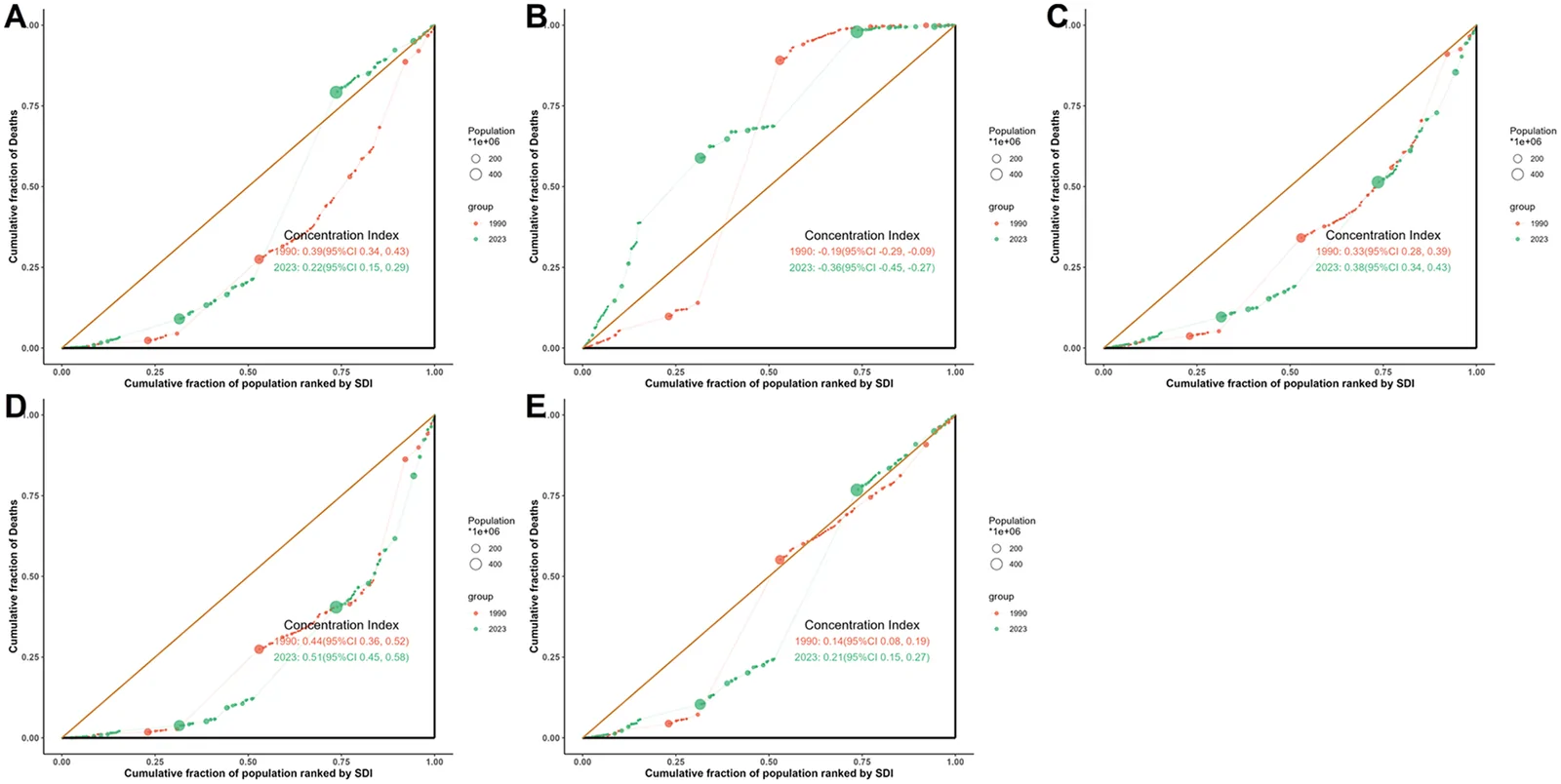
Health concentration curve for the cumulative deaths of female TBL cancer attributable to ambient particulate matter pollution **(A)**, household air pollution from solid fuels **(B)**, residential radon **(C)**, smoking **(D)**, secondhand smoke **(E)** globally, 1990-2023.

The concentration index of cumulative female TBL cancer mortality due to ambient particulate matter decreased from 0.39 (0.34, 0.43) in 1990 to 0.22 (0.15, 0.29) in 2023, while that due to household solid fuel use decreased from −0.19 (−0.29, −0.09) to −0.36 (−0.45, −0.27) (Figures 6A,B). In contrast, the concentration indices for radon, smoking, and secondhand smoke-attributed mortality increased from positive levels in 1990 to higher levels in 2023 (Figures 6C–E). The concentration index of cumulative DALY followed a comparable pattern (Supplementary Figure S6).

### Drivers and projections of burden by air pollution, radon, and tobacco exposure

**Figure 7:**
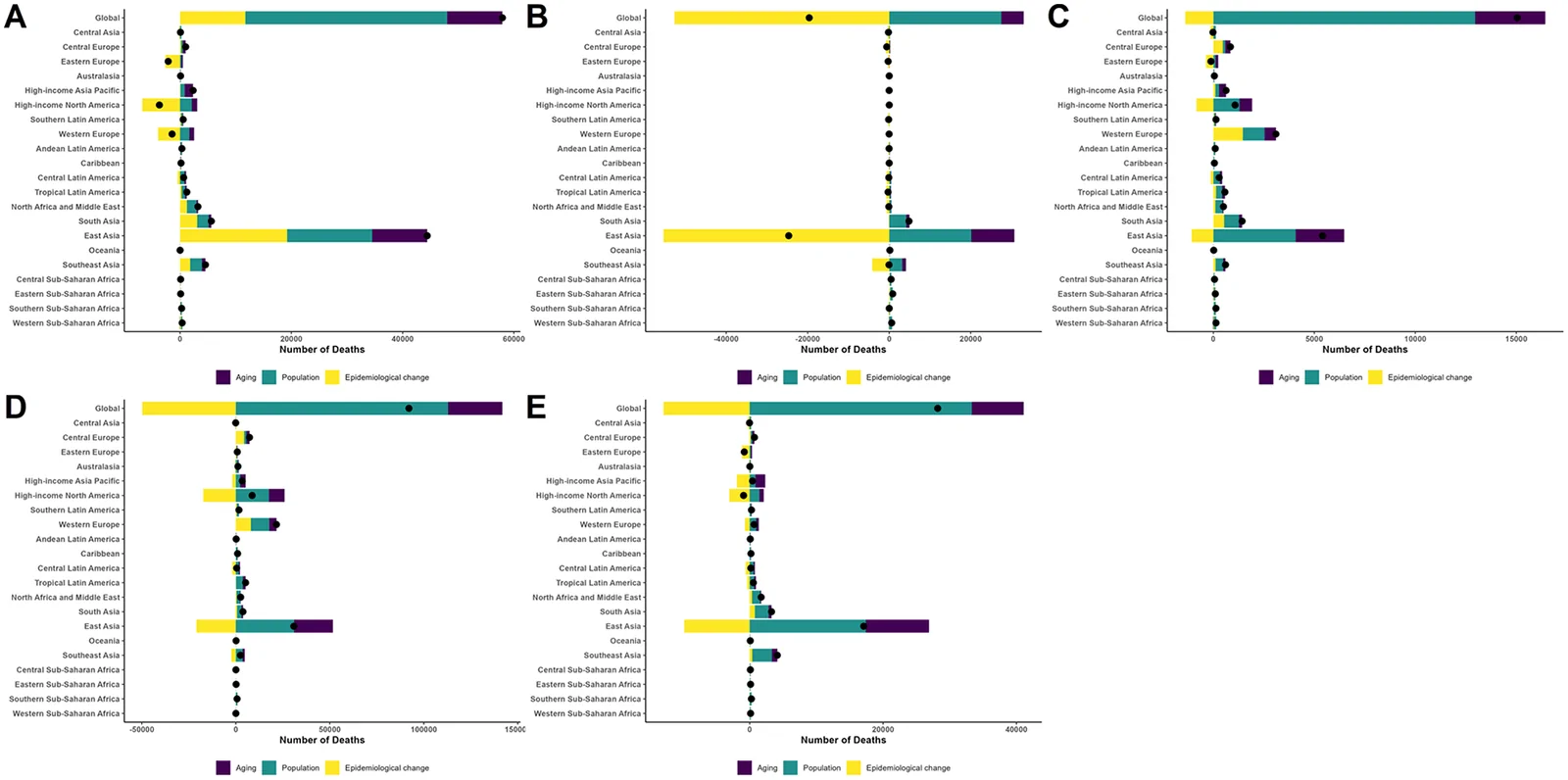
Driving factors of death number changes in female TBL cancer attributable to ambient particulate matter pollution **(A)**, household air pollution from solid fuels **(B)**, residential radon **(C)**, smoking **(D)**, secondhand smoke **(E)**, 1990-2023, by regions.

From 1990 to 2023, population growth and aging drove increases in female TBL cancer deaths attributed to ambient particulate matter, radon, smoking, and secondhand smoke worldwide; however, deaths attributed to solid fuel use declined because of negative contributions from epidemiological changes (Figure 7). The contributions of population growth and aging to increases in deaths were especially pronounced in East Asia (Figure 7). The epidemiological change-driven component of deaths due to ambient particulate matter increased globally and in East Asia, Southeast Asia, South Asia, and the Middle East and North Africa, but dropped in Western and Eastern Europe and high-income North America (Figure 7A). Parallel patterns were evident in the DALY decomposition analysis (Supplementary Figure S7).

**Figure 8:**
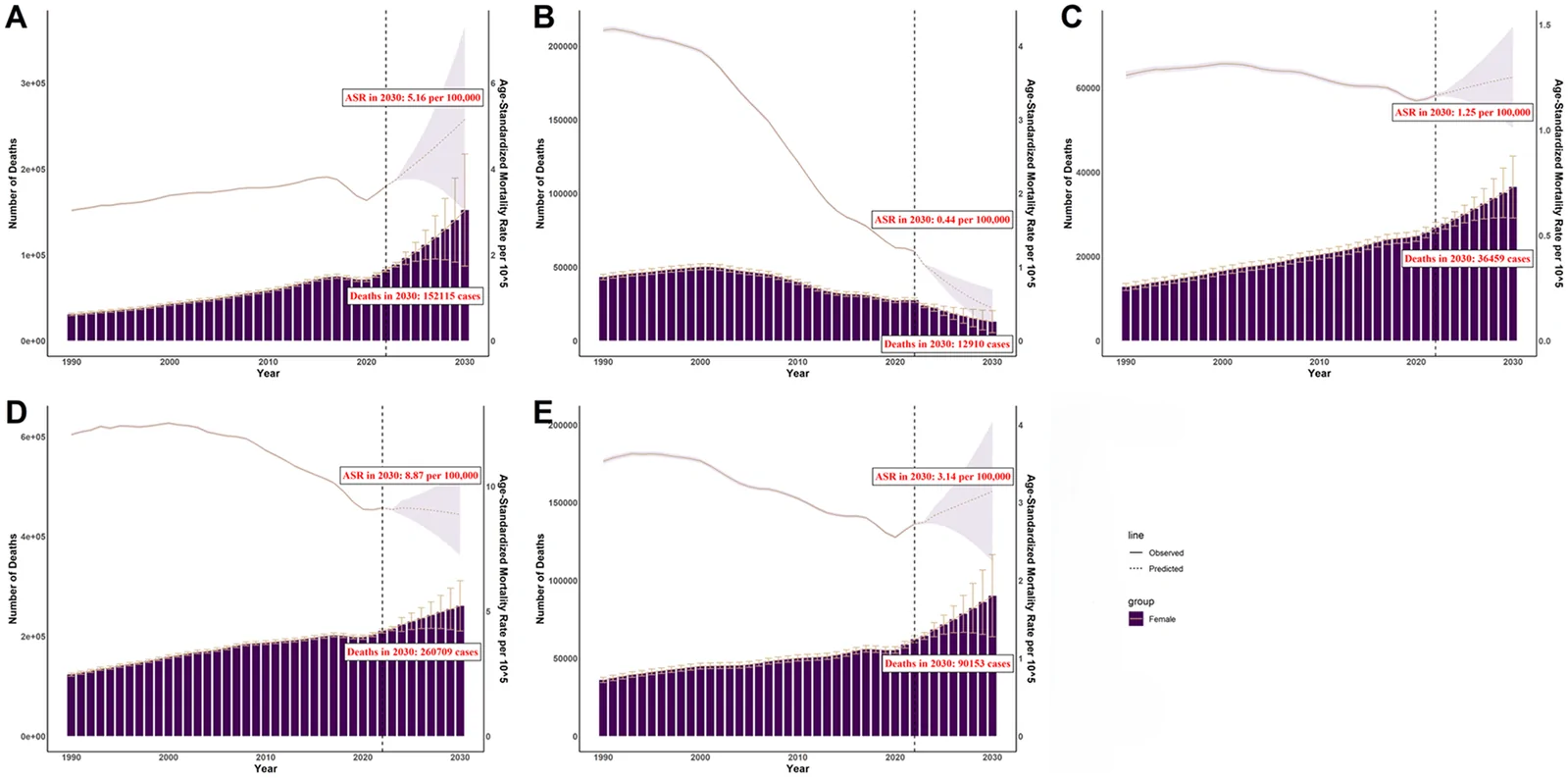
Predicted deaths and ASMR of female TBL cancer attributable to ambient particulate matter pollution **(A)**, household air pollution from solid fuels **(B)**, residential radon **(C)**, smoking **(D)**, secondhand smoke **(E)** globally, 1990-2030.

From 2024 to 2030, according to BAPC projections, women's TBL cancer deaths and ASMR attributable to ambient particulate matter, radon, and secondhand smoke were expected to continue increasing; however, those attributable to household solid fuel use were expected to decline (Figures 8A–C,8E). Smoking-attributable burden showed a divergent trend, with a slight decline in ASMR alongside a continued rise in deaths (Figure 8D). The predicted trends for DALYs and ASDR also demonstrated similar trends (Supplementary Figure S8).

## Discussion

Extensive GBD literature has rigorously explored TBL cancer, yet comprehensive analyses specifically addressing the burden of female TBL cancer attributable to inhalation-related risk factors remain limited (2–22). Our study shows that, despite an overall decline in the mortality and DALY rates of female TBL cancer attributable to inhalation-related exposures over the past 34 years, the burden has remained substantial and unevenly distributed across regions. Notably, ambient particulate matter pollution emerged as the only major inhalation-related risk factor showing a globally sustained increase in attributable burden over the past 34 years. In contrast, the burden from other exposures, although declining in higher-SDI regions, has continued to rise across nearly all attribution types in lower-SDI areas. Despite the consistently increasing trend of ambient particulate matter, smoking remained the leading contributor in 2023.

In the present study, we focused on five inhalation-related exposures, each of which is defined in the GBD framework as distinct from occupational or dietary risk factors. Together, these exposures represent routine inhalation-related risk factors. This integrated approach provides a comprehensive assessment of routine inhalation-related exposures within the GBD framework, allowing for estimation of the burden attributable to multiple exposures and facilitating interregional and sociodemographic comparisons. Importantly, such a framework also informs public health interventions that aim at multiple concurrent exposures holistically rather than in a piecemeal fashion. Earlier studies have likewise merged smoking (behavioral risk) and occupational asthmagens (occupational risk) from the GBD 2019 dataset to build a model for environment-related asthma, revealing regional and gender disparities (23). Consistent with previous studies, the present investigation leverages the newly released GBD 2023 dataset, encompassing a broader spectrum of environmental determinants, We focused on living-environment exposures and excluded occupational contexts. Women's TBL cancer burden, although lower than that in men, remains an important public health concern (24). Although some observed changes in ASMR and ASDR were modest in magnitude, even small variations in age-standardized rates may translate into substantial public health impact due to population growth and ageing, particularly in high-burden regions.

We found ambient particulate matter pollution to be a major contributor to female TBL cancer in 2023, second to tobacco smoking. Notably, it has stood out as the only major inhalation-related risk factor with a continuous upward trend across countries spanning the socio-demographic spectrum over 34 years, with particularly pronounced rises in populous regions, including China, India and Indonesia. After adjustment for demographic factors, pure epidemiological change remains positive, particularly in East Asia, but negative in Western countries. In addition, although the concentration index for ambient particulate matter-related female TBL cancer has remained positive over 34 years, indicating a sustained clustering of the burden in more developed regions, its absolute value has declined, suggesting that the burden is gradually concentrated in transitional economies rather than highly developed nations. This pattern is also supported by the unusually steep rise in particulate matter-related burden in middle-to-high SDI regions during this time. Observation shows that China is markedly elevated in the 2023 LOESS analysis, with a burden surpassing the level predicted by its SDI, suggesting a relatively higher observed burden compared with expectations based on SDI.

These patterns may be explained by a combination of socio-environmental factors. First, industrialization and urbanization in developing countries undergoing industrial transition have fueled sustained increases in particulate matter emissions, whereas mature economies have realized effective air quality management, which may partly explain the divergent trends observed in our analyses. Second, prolonged low-level exposure to fine particulate matter produces health effects that are difficult to reverse, with women potentially more affected due to differences in long-term exposure patterns and other susceptibility factors, although these mechanisms were not directly evaluated in this study. Third, fine particulate matter may be more strongly associated with higher TBL cancer risk among long-term never-smokers by reaching distal alveolar regions and initiating cancers in cell lineages less affected by tobacco, consistent with cohort studies showing elevated TBL cancer risk among women with no smoking history over ten years (25). These findings highlight the importance of considering targeted interventions, particularly for non-smoking women in mid-stage urbanizing economies of South, Southeast, and East Asia (26). Such interventions may include strengthening air-quality management, reducing particulate matter emissions, and expanding environmental monitoring programs in rapidly urbanizing regions.

We observed that in 2023, tobacco smoking accounted for the largest share of female TBL cancer burden, while secondhand smoke ranked third. Evidence suggests that the impact of secondhand smoke on non-smoking women living with smokers far exceeds the risk observed in smoke-free households (27). This suggests a higher burden of secondhand smoke among non-smoking women and highlights the importance of continued tobacco control measures to reduce both active and passive smoking exposure. Our findings suggest that the burden of female TBL cancer attributable to smoking tends to increase with national SDI, whereas secondhand smoke burden declines in more developed countries, which may reflect reductions in secondhand smoke exposure following tobacco control policies, although the burden from active smoking remains high. In Western and Central Europe, the increase in smoking-related female TBL cancer may contribute to the observed burden growth in some high-income regions, despite declining tobacco prevalence, which may reflect delayed effects of tobacco control measures (28). Deviation analyses similarly indicate that countries such as Hungary bear a smoking burden far exceeding SDI-based expectations. This may partly explain the observed increase in the concentration index for smoking burden over the past 34 years. By contrast, the rising concentration index for secondhand smoke predominantly reflects growing burdens in specific low-income yet high-potential East and Southeast Asia, where female smoking prevalence remains substantially lower than in Western countries, suggesting that non-smoking women in these regions may represent an important population for further risk assessment (29). These regional differences suggest that tobacco control strategies may need to be tailored across regions, with emphasis on reducing active smoking in Western-Central Europe and secondhand smoke exposure in East and Southeast Asia. By 2030, even if rates of smoking-related burden slightly decline, absolute deaths and DALYs from both active and secondhand smoking are expected to rise, highlighting population growth and aging. These findings support continued investment in comprehensive tobacco-control programs, including smoking cessation services, tobacco taxation, public education campaigns, and smoke-free policies aimed at reducing secondhand smoke exposure.

Although usually perceived as a dermal or contact hazard, radon exposure is primarily considered to occur via inhalation. As reported in this study, female TBL cancer attributable to radon, although representing a relatively small component of the overall inhalation-related burden, has shown a modest increase in less-advantaged regions with rapidly expanding populations over the past 34 years, consistent with the demographic contributions identified in the decomposition analysis. The concentration index for residential radon burden has exhibited a gradual escalation, suggesting a relative concentration of burden in higher-income countries, underscoring the importance of controlling indoor radon levels alongside economic progress. Central European nations, illustrated by Hungary, whose radon burden significantly surpasses LOESS predictions in 2023, face compounded risk from both radon and smoking, pointing to the dual exposure challenge. According to a European multicountry study, residential radon poses a significant carcinogenic risk for current smokers and recent quitters, suggesting that radon mitigation and tobacco control may be particularly relevant in these settings (30). Conversely, in Central Asia and Eastern Europe, areas that have undergone far-reaching societal transformations similar to those in Central Europe, female TBL cancer burden attributed to radon has declined over 34 years. This disparity, despite shared historical backgrounds, highlights the importance of context-specific public health strategies to address residential radon contamination. From an environmental health perspective, radon exposure reduction may require building-level interventions, including improved ventilation, radon-resistant construction, and routine indoor radon monitoring.

Our study shows that household solid fuel use has exhibited the fastest decline in the female TBL cancer burden among five inhalation-related risk factors during the past 34 years, and, despite population growth and ageing, the burden maintains a declining trajectory with forecasts suggesting the downward trend will persist until 2030. Later-stage economies, particularly in the Middle East, have played a central role in this reduction, with the solid fuel use-related burden falling sharply from peak levels in high-middle SDI areas. This finding aligns with prior evidence of a 70.6% reduction in exposure to household air pollution from solid fuels in this region in recent years (31). Yet alarmingly, economically disadvantaged countries in the Pacific Islands and sub-Saharan Africa remain afflicted by unfavorable patterns, with concentration indices becoming increasingly negative and highlighting that the burden is disproportionately concentrated in low-SDI settings, thereby underscoring the heightened public health challenge posed by the incomplete transition away from solid fuels. In particular, the related burden in the Solomon Islands and Papua New Guinea surpasses SDI-based expectations. The challenge of clean energy adoption in Pacific insular nations may be partly related to geographic fragmentation and higher logistical costs, which hinder the shift from traditional solid fuels (32). Conditions in sub-Saharan Africa appear more concerning, especially in central and eastern parts such as Ethiopia, where female TBL cancer burden from solid fuel use has surged, and other inhalation-related exposures have intensified to varying degrees; hampered by severely limited data, these findings should be interpreted with caution given potential data limitations. Consequently, economically lagging regions may benefit from strengthening surveillance systems in line with the goal of Universal Health Coverage (33). Nonetheless, policies in less-industrialized countries may be more likely to be adapted from existing international guidelines, rather than being flexibly tailored to local capacity and context, as often practiced in developed countries (34). Strengthening clean energy adoption through subsidies, infrastructure development, and targeted household-level interventions may help reduce reliance on solid fuels in resource-limited regions.

Amid a well-established GBD evidence base, the present study applies several established analytical methods within the GBD framework. First, by spanning a 34-year period and leveraging the latest GBD 2023 data, our research offers an up-to-date, long-horizon perspective on women's TBL cancer attributable to inhalation-related exposures, with a focus on female populations in a field historically dominated by studies of male populations. Next, this study provides an integrated assessment of inhalation-related exposures within the GBD framework, illustrating that environmental mitigation, including transitioning to clean fuels, reducing particulate matter, and controlling residential radon, and behavioral strategies, such as reducing active and secondhand smoking, both ultimately aim to reduce inhalation-mediated respiratory exposures and collectively represent a composite assessment of inhalation-related risk factors that provides evidence to support public health considerations. Additionally, informed by the methodological approaches of previous well-established studies, we incorporated decomposition techniques, concentration index analyses, deviation assessments, and predictive modeling to explore temporal trends, demographic contributions, and social disparities (35–39). Finally, by explicitly incorporating socioeconomic gradients, our analysis captures health disparities and delineates context-specific strategies across exposure-related TBL cancer burdens in diverse developmental landscapes, thereby facilitating policy-relevant interpretation of findings.

Notwithstanding these advantages, several inherent limitations warrant consideration. First of all, although GBD 2023 integrates multiple data sources to estimate disease burden, uncertainty may be greater in regions with sparse cancer registry data, where estimates rely more heavily on statistical modeling approaches. Besides, potential exposure misclassification may exist, as population-level exposure estimates cannot fully capture individual-level variability in environmental and behavioral risk factors. Furthermore, the quality and completeness of input data vary across countries and regions, which may introduce heterogeneity in burden estimation. At last, the comparative risk assessment (CRA) framework underlying GBD estimates relies on several assumptions, including exposure distributions and relative risk estimates derived from available epidemiological studies, which may introduce additional uncertainty in attributable burden estimates.

## Conclusions

This study advances current understanding by providing a comprehensive assessment of the substantial and uneven burden of female TBL cancer attributable to inhalation-related risk factors, primarily through inhalation-related pathways. Although the majority of the attributable burden has declined, the burden from ambient particulate matter continues to rise globally, while tobacco smoking remained the largest contributor in 2023. Our results highlight the need for coordinated, context-aware interventions targeting inhalation-related risk factors, especially in rapidly expanding populations undergoing profound economic transitions with relatively limited resources, to counter the mounting burden of female TBL cancer.

## Data Availability

Publicly available datasets were analyzed in this study. This data can be found here: Global Burden of Disease 2023 datasets (https://vizhub.healthdata.org/gbd-results/).
