# Supplementary material for: Trends, inequalities, and 2030 projections of women's tracheal, bronchial, and lung cancer attributable to air pollution, radon, and tobacco exposure
Source: Front Glob Womens Health. 2026 Aug 7;7:1662451. doi: 10.3389/fgwh.2026.1662451 (PMC13493495; doi:10.3389/fgwh.2026.1662451)
Supplement: Supplementary file 1 [file Datasheet1.pdf]

Socio-Demographic Index for 204 Countries and Territories from 1990 to 2023

location_name	1990	1991	1992	1993	1994	1995	1996	1997	1998	1999	2000	2001	2002	2003	2004	2005	2006	2007	2008	2009	2010	2011	2012	2013	2014	2015	2016	2017	2018	2019	2020	2021	2022	2023
Afghanistan	0.180215	0.180919	0.182439	0.183536	0.183155	0.183482	0.183144	0.182759	0.182583	0.182526	0.182386	0.182299	0.188052	0.195236	0.201865	0.208744	0.21588	0.223992	0.231763	0.240913	0.249932	0.258381	0.267013	0.275251	0.282874	0.289859	0.296511	0.302941	0.309144	0.315616	0.321745	0.32613	0.329956	0.333711
Albania	0.546515	0.544383	0.54176	0.540786	0.541369	0.543806	0.548082	0.551455	0.556702	0.563861	0.571675	0.580156	0.588205	0.595769	0.603155	0.610273	0.617236	0.623889	0.630319	0.63602	0.641443	0.646311	0.651012	0.655677	0.660671	0.665836	0.671107	0.676699	0.682273	0.687534	0.692002	0.697076	0.702067	0.706767
Algeria	0.449689	0.457182	0.464216	0.470606	0.476604	0.482645	0.489319	0.496321	0.503722	0.51164	0.520502	0.529503	0.538576	0.547527	0.555717	0.563606	0.570859	0.577493	0.583664	0.588821	0.594048	0.599219	0.603969	0.60832	0.612595	0.616811	0.621199	0.625795	0.630704	0.635888	0.64086	0.646329	0.651992	0.657757
American Samoa	0.612832	0.614772	0.617764	0.623121	0.629906	0.635409	0.638906	0.640878	0.641996	0.643543	0.645999	0.648488	0.651515	0.655521	0.660189	0.665398	0.669729	0.672266	0.672711	0.672587	0.67333	0.675205	0.677715	0.680686	0.684157	0.689184	0.695745	0.703041	0.711684	0.719878	0.727426	0.733557	0.73877	0.743031
Andorra	0.785967	0.790786	0.794095	0.796247	0.797732	0.798986	0.800611	0.802836	0.805137	0.807586	0.809874	0.813128	0.816829	0.822543	0.828119	0.832832	0.837507	0.841799	0.845618	0.848862	0.852477	0.856532	0.859747	0.861936	0.864244	0.866326	0.868305	0.870353	0.872325	0.874345	0.874986	0.876266	0.878145	0.879856
Angola	0.275845	0.280427	0.284803	0.287626	0.290457	0.293784	0.298253	0.302802	0.307553	0.312389	0.317041	0.321935	0.327864	0.333831	0.340467	0.348222	0.356967	0.366514	0.376559	0.385531	0.394875	0.404534	0.414321	0.423881	0.433345	0.442076	0.450158	0.458018	0.465696	0.473	0.479696	0.486315	0.493045	0.499297
Antigua and Barbuda	0.617134	0.623971	0.630028	0.635564	0.640528	0.644141	0.647933	0.652056	0.656471	0.660863	0.665542	0.669583	0.673625	0.678101	0.683047	0.688199	0.693855	0.699844	0.706047	0.71144	0.716176	0.720467	0.724491	0.727676	0.7304	0.732933	0.735636	0.738129	0.741162	0.744497	0.746087	0.748635	0.752078	0.755853
Argentina	0.611206	0.615339	0.621489	0.627489	0.633826	0.63881	0.643245	0.648494	0.654253	0.658043	0.661899	0.665397	0.667384	0.668495	0.670479	0.675055	0.678851	0.681254	0.683912	0.686574	0.69071	0.696099	0.700198	0.702465	0.705698	0.712519	0.719412	0.72636	0.735942	0.747186	0.754297	0.758912	0.762437	0.764786
Armenia	0.542994	0.545421	0.546581	0.549284	0.552973	0.557248	0.562058	0.567027	0.572461	0.577585	0.582891	0.588924	0.596169	0.604142	0.61213	0.620526	0.629131	0.638012	0.646149	0.651062	0.655788	0.660427	0.665535	0.670242	0.674566	0.679042	0.683254	0.688152	0.693251	0.698826	0.702879	0.707013	0.711995	0.717335
Australia	0.729689	0.733395	0.737321	0.74158	0.745705	0.74974	0.753898	0.758397	0.762868	0.767337	0.771588	0.775757	0.780168	0.784483	0.788056	0.791004	0.792789	0.794159	0.796806	0.800624	0.804768	0.808461	0.812416	0.817297	0.821754	0.825591	0.829335	0.832584	0.83626	0.840378	0.843867	0.847132	0.850137	0.852886
Austria	0.750364	0.752135	0.754878	0.759357	0.76534	0.770442	0.77509	0.779853	0.783947	0.787497	0.79143	0.794902	0.798049	0.800662	0.803333	0.806858	0.810504	0.813557	0.816475	0.818954	0.821672	0.825008	0.827901	0.830284	0.832383	0.83395	0.836031	0.839149	0.842458	0.845532	0.847616	0.849666	0.851831	0.85396
Azerbaijan	0.603847	0.603011	0.601207	0.5997	0.598123	0.595555	0.592579	0.589607	0.586794	0.585469	0.586136	0.588113	0.591366	0.59529	0.599522	0.606975	0.617954	0.629831	0.640148	0.647579	0.653724	0.659052	0.663637	0.66792	0.672538	0.676543	0.680316	0.684225	0.688345	0.692482	0.695815	0.699133	0.702281	0.705298
Bahamas	0.700954	0.696148	0.693962	0.700782	0.714285	0.726524	0.734787	0.74038	0.744317	0.746005	0.746282	0.746889	0.749086	0.752596	0.756185	0.759206	0.76155	0.764305	0.767932	0.771899	0.776327	0.780429	0.783902	0.786777	0.789878	0.792941	0.7958	0.798641	0.801567	0.804418	0.804812	0.8068	0.810078	0.813601
Bahrain	0.58272	0.58878	0.594402	0.600295	0.605995	0.611935	0.618342	0.624569	0.631078	0.638263	0.646366	0.65483	0.662189	0.668886	0.675246	0.681449	0.68748	0.693782	0.699948	0.704748	0.708493	0.711399	0.715259	0.720249	0.726075	0.732267	0.738685	0.745965	0.753419	0.760546	0.76677	0.772566	0.778332	0.784321
Bangladesh	0.23999	0.249141	0.257328	0.264333	0.271651	0.278167	0.283995	0.290973	0.297993	0.304209	0.31015	0.315326	0.319914	0.32462	0.33092	0.338055	0.345581	0.353731	0.362159	0.371068	0.38037	0.390795	0.40313	0.416325	0.429407	0.441521	0.452533	0.46285	0.473055	0.482469	0.490981	0.499428	0.508327	0.517107
Barbados	0.658693	0.661056	0.665296	0.670439	0.675319	0.67857	0.680409	0.681729	0.682902	0.683511	0.684536	0.686723	0.691204	0.696462	0.701189	0.704937	0.707956	0.710366	0.713107	0.716576	0.720615	0.724491	0.728432	0.731942	0.734511	0.73678	0.739222	0.741451	0.743812	0.746392	0.747731	0.749094	0.751783	0.754795
Belarus	0.627517	0.631949	0.637891	0.644041	0.650057	0.654822	0.659218	0.662588	0.664643	0.668169	0.673095	0.68005	0.686903	0.6938	0.701628	0.708464	0.714663	0.721379	0.729477	0.738044	0.746117	0.751066	0.755375	0.760522	0.765964	0.770857	0.776912	0.783824	0.789144	0.792734	0.795081	0.797968	0.800488	0.803626
Belgium	0.752859	0.757682	0.763626	0.769355	0.774623	0.778851	0.782623	0.786708	0.790215	0.793044	0.796431	0.800842	0.805122	0.808291	0.811443	0.814715	0.817637	0.820435	0.823352	0.826621	0.830711	0.83516	0.839814	0.844009	0.847618	0.85103	0.854653	0.858529	0.862006	0.865128	0.867737	0.870842	0.873878	0.876976
Belize	0.436579	0.446678	0.457403	0.468738	0.479216	0.488631	0.495612	0.500374	0.503483	0.506234	0.509729	0.513833	0.51887	0.525198	0.532357	0.539965	0.547649	0.55487	0.561024	0.566367	0.571144	0.575606	0.580072	0.584252	0.588409	0.592687	0.596961	0.601141	0.605747	0.610734	0.614157	0.61877	0.623596	0.628475
Benin	0.218748	0.222164	0.225623	0.22922	0.23256	0.236346	0.240024	0.243658	0.247131	0.250818	0.254826	0.258683	0.262571	0.266594	0.271081	0.275761	0.280599	0.285739	0.291117	0.296628	0.30229	0.308211	0.314692	0.321793	0.329467	0.337503	0.345635	0.353971	0.362621	0.371474	0.380082	0.388962	0.397851	0.406496
Bermuda	0.698556	0.702569	0.706331	0.710111	0.713603	0.717145	0.720598	0.724454	0.728678	0.733278	0.738343	0.743838	0.749008	0.754304	0.759602	0.764845	0.769129	0.772949	0.777249	0.781846	0.786634	0.79127	0.795702	0.799903	0.803679	0.806973	0.809797	0.812377	0.814833	0.817323	0.819872	0.822429	0.824798	0.82694
Bhutan	0.22852	0.236826	0.244176	0.250491	0.25745	0.265503	0.273899	0.282555	0.291266	0.300296	0.309349	0.3186	0.328339	0.338196	0.348061	0.357848	0.367739	0.378619	0.389602	0.401068	0.413204	0.425509	0.437704	0.449148	0.459866	0.470062	0.479914	0.489083	0.497168	0.504455	0.510119	0.515037	0.519682	0.524254
Bolivia (Plurinational State of)	0.428377	0.433829	0.439199	0.44492	0.451247	0.458049	0.465117	0.472248	0.47949	0.486235	0.492706	0.498914	0.504898	0.510838	0.516957	0.523083	0.529399	0.535614	0.541861	0.547745	0.553747	0.560043	0.566684	0.573695	0.580689	0.587498	0.594225	0.601045	0.607691	0.613792	0.618234	0.623033	0.627557	0.631803
Bosnia and Herzegovina	0.526897	0.527319	0.526008	0.523107	0.520645	0.520085	0.528039	0.540675	0.555792	0.571752	0.58569	0.597216	0.60719	0.615675	0.62347	0.630471	0.636801	0.642675	0.648839	0.654302	0.659221	0.663789	0.667963	0.672036	0.675723	0.679461	0.683349	0.687342	0.69154	0.695907	0.699728	0.704196	0.708729	0.712915
Botswana	0.439429	0.452407	0.464357	0.475578	0.486126	0.496653	0.507102	0.517578	0.527164	0.537248	0.547057	0.556079	0.564963	0.573509	0.581464	0.589432	0.597315	0.605128	0.612364	0.617639	0.62352	0.629441	0.63438	0.639574	0.644712	0.648568	0.65256	0.656454	0.660358	0.664092	0.666513	0.670065	0.673922	0.677582
Brazil	0.513149	0.519395	0.524974	0.530464	0.536041	0.541579	0.546942	0.552058	0.557002	0.562003	0.567586	0.573376	0.579397	0.585239	0.591333	0.597355	0.603555	0.610071	0.616714	0.622774	0.629108	0.635365	0.640947	0.646088	0.650685	0.654512	0.657728	0.661024	0.664555	0.668185	0.671569	0.675487	0.679602	0.68395
Brunei Darussalam	0.677063	0.682664	0.688412	0.694075	0.699682	0.705256	0.710958	0.716661	0.722666	0.728958	0.735468	0.742243	0.749093	0.755797	0.762166	0.767962	0.773111	0.777886	0.782249	0.786377	0.790256	0.793912	0.797758	0.801709	0.805639	0.809417	0.813006	0.816313	0.819279	0.822023	0.824696	0.827306	0.829884	0.832364
Bulgaria	0.624895	0.634886	0.643606	0.652031	0.660463	0.667219	0.67277	0.674727	0.673618	0.672694	0.676329	0.681606	0.686344	0.690109	0.6943	0.699035	0.703732	0.708618	0.713561	0.72018	0.727718	0.732996	0.736605	0.73932	0.742156	0.745256	0.747928	0.750555	0.753425	0.756809	0.759798	0.763471	0.767252	0.770862
Burkina Faso	0.133976	0.13749	0.140863	0.144216	0.147341	0.15072	0.154726	0.15898	0.163514	0.168323	0.172977	0.177879	0.182775	0.18788	0.192937	0.198219	0.203567	0.208832	0.214298	0.219787	0.225678	0.23183	0.238084	0.244557	0.251026	0.257469	0.264138	0.270825	0.277448	0.283909	0.289887	0.295864	0.301432	0.306761
Burundi	0.215077	0.218272	0.221219	0.223145	0.224741	0.225976	0.2264	0.226336	0.226801	0.227319	0.2273	0.227313	0.227948	0.228954	0.230776	0.232797	0.235416	0.238347	0.241865	0.245883	0.250496	0.255134	0.259875	0.265149	0.270785	0.275324	0.279485	0.283436	0.287512	0.291677	0.295429	0.29933	0.303253	0.30726
Cabo Verde	0.29183	0.2967	0.298735	0.302109	0.30979	0.319047	0.329808	0.341228	0.352824	0.36444	0.375976	0.386906	0.397339	0.407193	0.416571	0.425716	0.435131	0.444997	0.454393	0.463024	0.471445	0.479806	0.48746	0.494443	0.500773	0.506638	0.512754	0.519551	0.527195	0.535616	0.541838	0.548094	0.555368	0.56241
Cambodia	0.297912	0.301747	0.306471	0.311361	0.315706	0.320744	0.325701	0.330476	0.334887	0.340178	0.346047	0.352408	0.359238	0.366645	0.374663	0.383431	0.392322	0.401174	0.409703	0.417192	0.424049	0.430705	0.437279	0.443568	0.449742	0.45586	0.461918	0.467955	0.474058	0.480206	0.485193	0.490029	0.49496	0.499953
Cameroon	0.305169	0.311973	0.318143	0.323453	0.328131	0.332385	0.336345	0.340277	0.344054	0.347677	0.351282	0.354828	0.358302	0.362084	0.366315	0.370571	0.375267	0.380294	0.385382	0.390569	0.396084	0.401965	0.40813	0.414482	0.42123	0.42834	0.435715	0.443189	0.45077	0.458293	0.465513	0.472833	0.48008	0.487084
Canada	0.800709	0.802599	0.805162	0.807568	0.810258	0.814007	0.818388	0.822103	0.825323	0.829255	0.833485	0.837401	0.840911	0.844073	0.846773	0.848656	0.849998	0.851201	0.85287	0.855053	0.857877	0.860518	0.862962	0.86538	0.86788	0.870333	0.872665	0.875348	0.877991	0.880433	0.882366	0.884577	0.886259	0.887305
Central African Republic	0.203986	0.207076	0.209557	0.211582	0.213953	0.216278	0.217537	0.219156	0.220913	0.222738	0.224273	0.225731	0.227	0.22753	0.22808	0.228725	0.229871	0.231264	0.232843	0.234926	0.237439	0.240302	0.243796	0.242756	0.241452	0.24071	0.240765	0.241655	0.243175	0.245429	0.248113	0.251351	0.255015	0.259147
Chad	0.113535	0.117007	0.120447	0.123363	0.126261	0.128882	0.131048	0.133335	0.135735	0.137975	0.140064	0.142753	0.1456	0.149279	0.154902	0.160948	0.166478	0.171971	0.177075	0.181808	0.187206	0.192174	0.197317	0.201981	0.206772	0.211521	0.215361	0.218917	0.222478	0.226156	0.22949	0.232584	0.235866	0.239323
Chile	0.594355	0.600976	0.60691	0.612461	0.618042	0.624568	0.630837	0.637437	0.644989	0.652931	0.659763	0.666758	0.674414	0.680047	0.685008	0.689474	0.69284	0.695693	0.699174	0.703661	0.709723	0.716289	0.722713	0.728551	0.735092	0.743241	0.751002	0.757408	0.764105	0.771425	0.777379	0.782112	0.785434	0.788533
China	0.465309	0.473416	0.481463	0.489573	0.497648	0.506687	0.516988	0.527052	0.53585	0.544486	0.552336	0.558919	0.565621	0.572508	0.580648	0.589982	0.600578	0.611023	0.62082	0.630191	0.64216	0.652089	0.657128	0.661303	0.665078	0.667083	0.670197	0.678073	0.687058	0.696344	0.705673	0.71394	0.720965	0.727553
Colombia	0.495653	0.493833	0.493918	0.498289	0.506527	0.516135	0.52531	0.533999	0.54123	0.546627	0.551403	0.555812	0.560149	0.564564	0.569161	0.57411	0.579698	0.58661	0.593978	0.601206	0.608596	0.616039	0.623146	0.630251	0.637259	0.643938	0.650174	0.655971	0.661818	0.667829	0.672522	0.677841	0.683433	0.688855
Comoros	0.275714	0.284015	0.292295	0.300273	0.30723	0.314636	0.321608	0.328575	0.335096	0.341636	0.34811	0.354975	0.361967	0.368722	0.375273	0.382126	0.388796	0.394389	0.400058	0.405723	0.411093	0.41668	0.422393	0.428257	0.43393	0.43933	0.44482	0.450349	0.455769	0.460878	0.465567	0.470157	0.474547	0.478699
Congo	0.416123	0.422058	0.427177	0.431716	0.435161	0.438524	0.44219	0.445569	0.44879	0.451429	0.454941	0.458017	0.461483	0.464754	0.468177	0.472537	0.477449	0.48149	0.486325	0.491025	0.497339	0.504791	0.513502	0.5218	0.530359	0.53775	0.543823	0.549663	0.555343	0.560551	0.564898	0.569193	0.573535	0.577849
Cook Islands	0.58232	0.584934	0.588251	0.593006	0.600742	0.611015	0.623222	0.635685	0.646964	0.657002	0.66671	0.676369	0.684599	0.692299	0.699866	0.706067	0.712069	0.71656	0.719736	0.722313	0.72431	0.726766	0.731292	0.737308	0.743512	0.750052	0.755408	0.758968	0.762183	0.763833	0.764221	0.76649	0.771832	0.777405
Costa Rica	0.531632	0.536795	0.542155	0.547318	0.552645	0.557853	0.562552	0.567373	0.572235	0.577895	0.585092	0.592811	0.599092	0.603999	0.608797	0.612971	0.616359	0.61982	0.624552	0.630441	0.635679	0.640007	0.645508	0.651281	0.656717	0.662027	0.667637	0.673986	0.681865	0.691218	0.699112	0.705499	0.710629	0.715386
Coted'Ivoire	0.283947	0.29087	0.297695	0.303911	0.309254	0.313898	0.318339	0.322943	0.327669	0.332059	0.335645	0.338952	0.342158	0.345243	0.348096	0.350305	0.352155	0.354088	0.356501	0.359492	0.363288	0.366843	0.371449	0.377071	0.383541	0.390811	0.398191	0.40587	0.413759	0.42175	0.429277	0.436879	0.444364	0.451524
Croatia	0.659262	0.664971	0.668126	0.667977	0.667107	0.666399	0.668426	0.673977	0.679423	0.684063	0.689908	0.69578	0.701123	0.706298	0.711051	0.716358	0.721766	0.726207	0.73087	0.735883	0.740611	0.744077	0.746921	0.749809	0.752379	0.755044	0.757921	0.761	0.764011	0.767434	0.769783	0.77382	0.778077	0.782133
Cuba	0.582798	0.588327	0.590963	0.589821	0.587369	0.585094	0.583555	0.582764	0.583019	0.585514	0.590436	0.596608	0.603967	0.611701	0.618227	0.623616	0.628701	0.630938	0.632257	0.634283	0.637576	0.642654	0.648675	0.654452	0.659835	0.665571	0.670571	0.674394	0.678611	0.683084	0.686123	0.689317	0.692176	0.694881
Cyprus	0.64893	0.657552	0.668323	0.680012	0.691737	0.703223	0.71352	0.722973	0.732341	0.741521	0.750422	0.759081	0.767011	0.773823	0.780071	0.785795	0.791872	0.799242	0.806583	0.812553	0.817735	0.822178	0.825871	0.82853	0.830555	0.832425	0.834574	0.836971	0.839585	0.842349	0.844557	0.847888	0.851815	0.855816
Czechia	0.676023	0.682545	0.69193	0.705278	0.72068	0.732724	0.740661	0.746098	0.751364	0.756756	0.762037	0.766301	0.770952	0.77644	0.781044	0.785227	0.788744	0.79194	0.79533	0.798398	0.802092	0.805297	0.807351	0.808658	0.809347	0.80983	0.810612	0.812715	0.815918	0.819468	0.822559	0.826106	0.82894	0.831034
Democratic People's Republic of Korea	0.501894	0.503166	0.503517	0.503384	0.502982	0.502188	0.501205	0.499732	0.498663	0.498987	0.500008	0.502171	0.505035	0.508486	0.512498	0.517295	0.521865	0.526161	0.530845	0.535086	0.539064	0.542983	0.546988	0.551054	0.555099	0.558731	0.562861	0.566186	0.568581	0.570964	0.572453	0.573858	0.575232	0.57706
Democratic Republic of the Congo	0.291746	0.292907	0.293904	0.292978	0.290526	0.287768	0.284207	0.28001	0.275315	0.270755	0.265696	0.260908	0.257139	0.255448	0.25588	0.258156	0.261815	0.267138	0.273163	0.279214	0.286688	0.294696	0.303172	0.312264	0.321723	0.331303	0.340491	0.348833	0.356933	0.364783	0.371972	0.37937	0.38737	0.395104
Denmark	0.813355	0.816997	0.820783	0.824252	0.827927	0.831914	0.836201	0.840574	0.844542	0.84824	0.852148	0.856143	0.860086	0.863374	0.866227	0.868563	0.870792	0.872437	0.87426	0.876259	0.878873	0.881771	0.884343	0.886609	0.888308	0.889835	0.891866	0.895064	0.898935	0.902963	0.906233	0.909756	0.913073	0.91599
Djibouti	0.33445	0.338806	0.341541	0.34282	0.344122	0.346655	0.348753	0.351407	0.354082	0.356934	0.359559	0.362331	0.365136	0.368503	0.372305	0.376186	0.38101	0.385958	0.39138	0.396342	0.401079	0.406791	0.412841	0.419248	0.425801	0.432657	0.439389	0.446088	0.454162	0.462169	0.469583	0.476881	0.483987	0.491007
Dominica	0.548941	0.554788	0.563454	0.576357	0.591653	0.607035	0.619793	0.62974	0.637531	0.643896	0.650068	0.656094	0.661674	0.667408	0.673051	0.678266	0.683297	0.688291	0.693308	0.697676	0.701966	0.706187	0.710642	0.71585	0.721788	0.727543	0.733356	0.737439	0.741024	0.744772	0.746105	0.748169	0.750764	0.753664
Dominican Republic	0.447928	0.450543	0.453843	0.457818	0.462157	0.467008	0.472547	0.47911	0.486344	0.493961	0.501688	0.509397	0.517433	0.524992	0.532538	0.540221	0.54796	0.555495	0.562247	0.568504	0.57516	0.581556	0.587832	0.594114	0.600423	0.606999	0.613835	0.620427	0.627247	0.634061	0.639525	0.645873	0.652159	0.658091
Ecuador	0.534758	0.534385	0.535224	0.537611	0.54185	0.546152	0.54956	0.552282	0.554403	0.556566	0.561161	0.567453	0.57417	0.580224	0.585295	0.589152	0.591838	0.594276	0.597514	0.601699	0.607581	0.615006	0.623253	0.631228	0.637786	0.64308	0.647775	0.653204	0.660015	0.667817	0.674596	0.680834	0.686265	0.690997
Egypt	0.437312	0.445967	0.455255	0.463797	0.471923	0.480002	0.488214	0.496466	0.504809	0.512743	0.520865	0.529145	0.5371	0.544687	0.551683	0.557669	0.562481	0.566003	0.568479	0.569915	0.571563	0.574062	0.578686	0.58523	0.593167	0.602052	0.611267	0.620396	0.629716	0.639012	0.648212	0.657659	0.667281	0.67652
El Salvador	0.394901	0.397335	0.400529	0.404573	0.409456	0.415573	0.422347	0.42984	0.438336	0.447787	0.45789	0.467608	0.476206	0.483884	0.490775	0.497414	0.503687	0.509391	0.514993	0.520521	0.526446	0.532942	0.539507	0.546339	0.553309	0.560395	0.567584	0.574546	0.581234	0.58758	0.592364	0.59782	0.603036	0.60814
Equatorial Guinea	0.269189	0.275909	0.28413	0.292776	0.300927	0.31029	0.323742	0.348896	0.368755	0.388004	0.407622	0.428874	0.446103	0.461043	0.476023	0.48991	0.501848	0.513488	0.525618	0.536027	0.545506	0.555552	0.565906	0.575621	0.58515	0.593717	0.601493	0.609052	0.616269	0.622872	0.629071	0.635015	0.641044	0.646242
Eritrea	0.218731	0.225297	0.233845	0.244056	0.256661	0.26859	0.280831	0.292926	0.303936	0.313667	0.320812	0.327811	0.333803	0.338224	0.341789	0.344889	0.347318	0.349447	0.349219	0.350142	0.352524	0.3581	0.363724	0.367663	0.375496	0.379562	0.384379	0.38763	0.392292	0.397002	0.4012	0.405461	0.409949	0.414533
Estonia	0.673915	0.684019	0.69453	0.702888	0.70774	0.712509	0.718269	0.724269	0.728658	0.732937	0.739441	0.746346	0.752353	0.757497	0.762907	0.768771	0.774036	0.780591	0.788049	0.794269	0.800522	0.806318	0.810731	0.814509	0.818532	0.823035	0.827295	0.831041	0.834867	0.839605	0.844244	0.848814	0.852244	0.85486
Eswatini	0.398761	0.40834	0.41753	0.426375	0.434785	0.443073	0.450851	0.45764	0.463661	0.469718	0.476016	0.481905	0.487459	0.492802	0.498071	0.503667	0.509558	0.515505	0.52145	0.527701	0.534102	0.540345	0.546755	0.553348	0.559537	0.565415	0.571026	0.576439	0.581608	0.586377	0.590946	0.596565	0.601725	0.606902
Ethiopia	0.137575	0.140182	0.141941	0.144201	0.146223	0.148759	0.152019	0.155929	0.159267	0.163067	0.167347	0.172616	0.177671	0.182505	0.189081	0.197104	0.205915	0.216352	0.227593	0.239301	0.251602	0.264313	0.276834	0.288967	0.301014	0.312593	0.323926	0.334752	0.345295	0.356175	0.366353	0.375989	0.385059	0.393768
Fiji	0.540244	0.545117	0.550371	0.555743	0.561415	0.567427	0.574015	0.580065	0.586052	0.592423	0.598019	0.603216	0.608026	0.612367	0.616613	0.620119	0.623501	0.626347	0.628978	0.631132	0.633733	0.636823	0.640071	0.643912	0.648697	0.653798	0.65875	0.663843	0.669011	0.673863	0.676432	0.678432	0.682226	0.686419
Finland	0.768828	0.771522	0.774526	0.777903	0.782007	0.786576	0.790673	0.795164	0.799092	0.802999	0.80766	0.812504	0.817222	0.821086	0.824771	0.828382	0.831821	0.835631	0.839211	0.842124	0.845805	0.849564	0.852989	0.856259	0.859578	0.863341	0.867432	0.87175	0.875613	0.879052	0.881945	0.885121	0.888241	0.890815
France	0.742695	0.748762	0.755508	0.761577	0.766423	0.770666	0.774677	0.778611	0.782066	0.784741	0.787442	0.790998	0.795026	0.798262	0.801241	0.804287	0.80758	0.810817	0.813476	0.81579	0.818563	0.821842	0.825132	0.828549	0.832202	0.836053	0.839667	0.843142	0.846259	0.849559	0.852488	0.855609	0.85841	0.861099
Gabon	0.423449	0.432759	0.441626	0.450506	0.459439	0.468478	0.477135	0.485449	0.493288	0.500241	0.506913	0.512783	0.518086	0.523007	0.527842	0.533026	0.537157	0.541442	0.54538	0.548757	0.552869	0.557537	0.562283	0.567192	0.572428	0.578066	0.583904	0.590005	0.596486	0.603463	0.610349	0.617271	0.624387	0.631086
Gambia	0.246661	0.253016	0.259064	0.264853	0.270176	0.275253	0.280348	0.285454	0.290893	0.296833	0.302961	0.309101	0.314293	0.319575	0.325546	0.330955	0.335708	0.340514	0.345463	0.35083	0.356757	0.361879	0.36766	0.373689	0.379265	0.385101	0.39065	0.39605	0.401891	0.407866	0.413675	0.419898	0.426266	0.432615
Georgia	0.707436	0.711905	0.714993	0.716148	0.715283	0.713312	0.710821	0.708293	0.706076	0.705196	0.70635	0.709314	0.712992	0.716567	0.719623	0.722518	0.726154	0.731333	0.736698	0.741582	0.746762	0.751505	0.755887	0.759605	0.76347	0.76804	0.77342	0.779062	0.7849	0.790753	0.795232	0.799745	0.804416	0.808808
Germany	0.796604	0.802724	0.807588	0.811618	0.814892	0.816845	0.81839	0.820549	0.822892	0.825107	0.827613	0.830452	0.833195	0.83551	0.837784	0.840116	0.842784	0.845744	0.848832	0.851063	0.853633	0.856331	0.858567	0.860334	0.861537	0.862083	0.863532	0.866435	0.869055	0.87151	0.873425	0.875218	0.876918	0.878636
Ghana	0.374661	0.381431	0.387907	0.393947	0.399796	0.405567	0.411359	0.417039	0.422419	0.427624	0.432915	0.43783	0.442728	0.447737	0.452699	0.457685	0.462796	0.46834	0.474587	0.480729	0.486967	0.494216	0.501613	0.508926	0.515705	0.5217	0.527494	0.533634	0.540141	0.546998	0.553454	0.560243	0.567069	0.573769
Greece	0.683848	0.690744	0.698338	0.705443	0.712049	0.718469	0.72443	0.730603	0.737167	0.743671	0.749269	0.754181	0.759464	0.765063	0.770658	0.774971	0.779188	0.783385	0.78759	0.79194	0.796501	0.800481	0.803408	0.80521	0.806306	0.806922	0.807762	0.809685	0.81212	0.815037	0.817847	0.822192	0.826879	0.831148
Greenland	0.764172	0.762416	0.761278	0.761367	0.761931	0.760665	0.75857	0.758644	0.761195	0.76354	0.766745	0.77148	0.77847	0.784897	0.79018	0.796524	0.803572	0.808319	0.811763	0.816688	0.82264	0.827576	0.830596	0.832913	0.834554	0.834963	0.835119	0.835366	0.836629	0.840476	0.846995	0.853193	0.857511	0.860114
Grenada	0.467236	0.478503	0.49014	0.501312	0.512263	0.522504	0.532274	0.54131	0.550309	0.558676	0.566344	0.572867	0.578878	0.585146	0.590694	0.596978	0.60205	0.607309	0.612213	0.616184	0.620001	0.623787	0.627507	0.631321	0.635912	0.641066	0.646469	0.651921	0.657355	0.662527	0.665926	0.669723	0.673875	0.678048
Guam	0.678722	0.671854	0.666098	0.663916	0.666393	0.67281	0.682328	0.693695	0.705385	0.716556	0.72627	0.734188	0.739804	0.742835	0.744743	0.74565	0.745716	0.746262	0.747063	0.746849	0.746591	0.747398	0.750425	0.755095	0.760859	0.766555	0.772067	0.778321	0.785206	0.792691	0.798967	0.804617	0.808773	0.811869
Guatemala	0.322004	0.32427	0.329311	0.336488	0.342586	0.348368	0.355521	0.365568	0.377497	0.385455	0.389374	0.395504	0.405308	0.416535	0.427793	0.437747	0.445928	0.453682	0.461791	0.46813	0.473458	0.47919	0.483781	0.48835	0.49407	0.500033	0.506349	0.513074	0.520731	0.528915	0.535858	0.542281	0.548173	0.553758
Guinea	0.182976	0.185893	0.189114	0.192449	0.195916	0.199783	0.204341	0.20896	0.213839	0.21889	0.223791	0.228189	0.232479	0.236806	0.241256	0.245553	0.249445	0.253456	0.257834	0.262101	0.26703	0.272363	0.278128	0.284115	0.290159	0.296244	0.302811	0.310405	0.31782	0.325378	0.332966	0.340584	0.347998	0.355151
Guinea-Bissau	0.209362	0.214021	0.218361	0.222864	0.227451	0.232094	0.236918	0.242193	0.24533	0.249207	0.253094	0.257026	0.260907	0.264669	0.268536	0.272634	0.276624	0.280837	0.285424	0.290018	0.295082	0.300905	0.306195	0.311609	0.316913	0.322954	0.329417	0.336232	0.342701	0.348928	0.354813	0.361037	0.367279	0.373342
Guyana	0.456822	0.459643	0.463824	0.469037	0.475165	0.482005	0.489952	0.49887	0.50746	0.516177	0.523779	0.530745	0.537334	0.543387	0.549403	0.554851	0.560565	0.566362	0.572019	0.577935	0.584047	0.59036	0.596642	0.602829	0.608687	0.614719	0.620637	0.62672	0.632945	0.639334	0.649483	0.660659	0.677736	0.69558
Haiti	0.322011	0.326908	0.331697	0.336161	0.33961	0.344072	0.349104	0.35451	0.360244	0.366446	0.372827	0.378749	0.38445	0.390025	0.395031	0.399878	0.404486	0.409448	0.414136	0.419167	0.423423	0.428055	0.432191	0.436393	0.440465	0.444489	0.448154	0.451564	0.454842	0.457572	0.459697	0.461432	0.462761	0.463795
Honduras	0.334907	0.339233	0.343746	0.348513	0.353181	0.358293	0.36323	0.368264	0.374017	0.38021	0.387321	0.39453	0.401981	0.409555	0.417113	0.424517	0.431887	0.438942	0.445648	0.45155	0.457423	0.463314	0.469019	0.474058	0.478771	0.483186	0.48708	0.491223	0.495868	0.501026	0.505111	0.510165	0.5152	0.520269
Hungary	0.645457	0.649759	0.656714	0.663842	0.670968	0.678681	0.686205	0.693175	0.699504	0.705167	0.710872	0.717019	0.723154	0.729079	0.734494	0.739572	0.744454	0.748621	0.752779	0.756904	0.760668	0.762961	0.76396	0.764768	0.765931	0.767497	0.769679	0.772733	0.775761	0.778678	0.781338	0.785457	0.789943	0.79361
Iceland	0.767421	0.772134	0.775813	0.779697	0.783437	0.786334	0.788898	0.792021	0.79591	0.800474	0.806191	0.812149	0.816852	0.820166	0.82306	0.826131	0.829439	0.833319	0.837698	0.841514	0.844978	0.848223	0.850933	0.853984	0.857609	0.862026	0.866394	0.870425	0.874388	0.878089	0.880521	0.883241	0.886162	0.888966
India	0.341586	0.347095	0.352581	0.35824	0.364222	0.370676	0.37745	0.384049	0.390755	0.397643	0.404189	0.410407	0.4163	0.42242	0.428873	0.435759	0.443198	0.451148	0.45891	0.467266	0.476477	0.486241	0.496789	0.508068	0.519809	0.531793	0.543662	0.554886	0.565496	0.575197	0.583043	0.591103	0.599032	0.606982
Indonesia	0.460321	0.469124	0.477936	0.486532	0.494891	0.502959	0.510766	0.518495	0.524277	0.529255	0.533621	0.537725	0.542059	0.546551	0.551049	0.555635	0.5605	0.566123	0.572708	0.579583	0.58694	0.594739	0.602307	0.609559	0.6165	0.623065	0.629309	0.635278	0.641002	0.646622	0.651397	0.65608	0.660892	0.665732
Iran (Islamic Republic of)	0.453887	0.472034	0.486368	0.497954	0.508874	0.519988	0.530758	0.540356	0.549503	0.559302	0.569742	0.580071	0.590392	0.600679	0.610996	0.621242	0.629747	0.636315	0.641062	0.645154	0.649856	0.654973	0.65873	0.66181	0.665275	0.669111	0.674374	0.680537	0.687185	0.693351	0.699235	0.705215	0.711359	0.717585
Iraq	0.433403	0.434484	0.436152	0.439421	0.442439	0.444425	0.447563	0.452211	0.459848	0.468561	0.477315	0.4851	0.490928	0.492826	0.497999	0.502727	0.50738	0.511669	0.517049	0.523674	0.531616	0.540812	0.551428	0.562368	0.572435	0.581624	0.591555	0.601328	0.611017	0.620799	0.629021	0.637323	0.645957	0.653813
Ireland	0.745034	0.750579	0.7576	0.764361	0.770216	0.775255	0.780513	0.786579	0.793119	0.800266	0.807048	0.813425	0.820351	0.827442	0.834262	0.839129	0.841918	0.844456	0.847427	0.851319	0.855788	0.859765	0.863471	0.866952	0.870578	0.876292	0.881777	0.887347	0.893676	0.897402	0.900453	0.902346	0.90448	0.906907
Israel	0.729507	0.733762	0.738485	0.743064	0.747685	0.752198	0.756272	0.759409	0.76214	0.765311	0.76937	0.77307	0.77609	0.778972	0.782494	0.78598	0.789225	0.791289	0.792434	0.793657	0.795742	0.798482	0.80144	0.80423	0.806835	0.80936	0.812184	0.815587	0.819781	0.824369	0.82808	0.832071	0.836675	0.84093
Italy	0.722882	0.728583	0.734216	0.739709	0.745209	0.75026	0.754899	0.759135	0.762478	0.765623	0.769388	0.773399	0.777173	0.78014	0.782821	0.785684	0.78834	0.790607	0.792995	0.795518	0.798264	0.800948	0.803431	0.805786	0.80804	0.810491	0.813112	0.816071	0.819276	0.822545	0.824967	0.82808	0.83126	0.834439
Jamaica	0.546359	0.551498	0.557477	0.564009	0.57065	0.577396	0.583875	0.589819	0.595184	0.600332	0.605354	0.610435	0.615513	0.620851	0.625717	0.63024	0.634749	0.63911	0.643444	0.647542	0.651324	0.655221	0.659105	0.662717	0.666156	0.6696	0.672968	0.6761	0.679043	0.681676	0.68305	0.6853	0.688451	0.691834
Japan	0.795726	0.800019	0.804355	0.808111	0.811613	0.815336	0.818547	0.821031	0.823122	0.824947	0.826469	0.82807	0.830139	0.832369	0.834785	0.836923	0.83859	0.840386	0.842259	0.843821	0.845906	0.848186	0.850242	0.852344	0.854475	0.856652	0.85893	0.861347	0.863871	0.866533	0.869011	0.871855	0.874008	0.875884
Jordan	0.552456	0.555841	0.559891	0.564048	0.568411	0.573057	0.577441	0.581838	0.586146	0.590371	0.594513	0.598679	0.603115	0.607657	0.612862	0.618552	0.624988	0.631997	0.639025	0.645944	0.652796	0.659653	0.666329	0.672176	0.677257	0.681977	0.68649	0.69121	0.696025	0.700854	0.705393	0.710164	0.715176	0.720352
Kazakhstan	0.595235	0.596182	0.600969	0.608275	0.615809	0.623563	0.630234	0.634149	0.637884	0.643167	0.647987	0.65171	0.654214	0.656857	0.660252	0.664244	0.668121	0.672037	0.676746	0.68237	0.687957	0.692123	0.695102	0.697939	0.702011	0.706321	0.71024	0.713434	0.715033	0.716214	0.716912	0.720077	0.723951	0.727191

Kenya	0.33845	0.34542	0.351535	0.357016	0.362154	0.366779	0.371219	0.375194	0.378974	0.382558	0.385822	0.389207	0.392671	0.396323	0.400396	0.405071	0.410308	0.416294	0.422313	0.428753	0.436008	0.443733	0.451916	0.460298	0.468912	0.477816	0.486798	0.495864	0.505033	0.514153	0.522571	0.53141	0.540226	0.548711
Kiribati	0.428689	0.430259	0.433309	0.436503	0.439949	0.443597	0.447007	0.450273	0.454075	0.45745	0.461576	0.465036	0.468527	0.471788	0.47461	0.47755	0.480433	0.483331	0.485519	0.487608	0.489483	0.491695	0.494745	0.498614	0.502287	0.507267	0.51223	0.517011	0.521897	0.526349	0.529987	0.534348	0.538529	0.542689
Kuwait	0.687171	0.690125	0.689746	0.688344	0.687917	0.690403	0.696594	0.70742	0.719834	0.730279	0.73845	0.743843	0.748038	0.753083	0.759905	0.768443	0.776	0.784361	0.792482	0.799752	0.806027	0.811475	0.816608	0.821709	0.827292	0.833424	0.839753	0.84616	0.852332	0.858326	0.863922	0.868803	0.873106	0.877143
Kyrgyzstan	0.522835	0.526606	0.53064	0.53589	0.540468	0.54316	0.545739	0.547603	0.546935	0.545868	0.546124	0.546873	0.547183	0.547838	0.548698	0.549432	0.550912	0.552796	0.554725	0.556559	0.557216	0.559149	0.562261	0.567036	0.572338	0.577584	0.582445	0.588723	0.596409	0.604497	0.610631	0.616088	0.620955	0.625153
Lao People's Democratic Republic	0.279023	0.283408	0.287834	0.292415	0.297381	0.302617	0.30819	0.314286	0.320817	0.328248	0.336434	0.345696	0.355487	0.365368	0.375033	0.384582	0.394484	0.404469	0.413936	0.423126	0.431762	0.44001	0.447882	0.455487	0.462754	0.469682	0.476403	0.482891	0.489019	0.494969	0.500325	0.505412	0.510299	0.515008
Latvia	0.685036	0.692809	0.700912	0.710044	0.717362	0.722424	0.725852	0.728513	0.730129	0.731801	0.735472	0.740714	0.746159	0.752355	0.759316	0.765887	0.772265	0.780211	0.790032	0.798281	0.802037	0.803003	0.805523	0.807419	0.80852	0.811857	0.816344	0.822037	0.827526	0.831928	0.836673	0.842271	0.847089	0.850844
Lebanon	0.56351	0.566185	0.568952	0.571729	0.574691	0.578198	0.582619	0.5875	0.593145	0.598523	0.603319	0.607755	0.612198	0.616548	0.621614	0.626915	0.63191	0.637747	0.644365	0.652217	0.660314	0.66753	0.674471	0.681649	0.689041	0.695664	0.701141	0.706193	0.71108	0.715376	0.717356	0.71923	0.721274	0.723672
Lesotho	0.342861	0.348803	0.355084	0.361456	0.367821	0.373622	0.379412	0.385181	0.390455	0.395629	0.400808	0.4062	0.411329	0.416385	0.421141	0.425708	0.430346	0.435246	0.440727	0.445714	0.450941	0.456285	0.461834	0.467638	0.473712	0.479858	0.485717	0.490535	0.494708	0.49851	0.501281	0.504203	0.507045	0.509817
Liberia	0.246084	0.242257	0.241731	0.241526	0.237884	0.231879	0.224669	0.22151	0.22158	0.225455	0.235269	0.244708	0.254605	0.258337	0.261979	0.265951	0.270277	0.275048	0.280021	0.285092	0.290599	0.29684	0.304326	0.313066	0.321501	0.330018	0.338	0.345862	0.353944	0.360218	0.36564	0.371376	0.377254	0.383021
Libya	0.579795	0.593249	0.605739	0.617144	0.627964	0.637503	0.646863	0.655672	0.663625	0.670824	0.677843	0.684367	0.690301	0.697157	0.703945	0.711523	0.718805	0.72585	0.732585	0.738333	0.744253	0.744231	0.749109	0.751559	0.751727	0.751848	0.751898	0.754799	0.758788	0.761657	0.761804	0.76493	0.766947	0.770482
Lithuania	0.662181	0.666917	0.67449	0.681115	0.684985	0.68863	0.692485	0.696513	0.700864	0.706102	0.713085	0.720076	0.726531	0.73378	0.74114	0.748021	0.754228	0.761194	0.769728	0.778937	0.7871	0.792613	0.797522	0.802217	0.806594	0.811581	0.817632	0.824019	0.830106	0.836605	0.842818	0.848353	0.852608	0.856188
Luxembourg	0.773039	0.779045	0.783138	0.787731	0.793661	0.799504	0.80425	0.808811	0.813249	0.817135	0.819894	0.823461	0.826832	0.829955	0.832973	0.836222	0.840502	0.845066	0.848698	0.851638	0.854471	0.857675	0.86146	0.865322	0.869031	0.872805	0.876519	0.879828	0.882509	0.884851	0.88726	0.889841	0.892377	0.894757
Madagascar	0.272048	0.272513	0.272493	0.27211	0.271436	0.270783	0.270393	0.270526	0.27117	0.272136	0.273442	0.275466	0.27621	0.277819	0.279839	0.282171	0.285125	0.28856	0.292517	0.295876	0.299512	0.303702	0.308459	0.31353	0.31892	0.324631	0.33103	0.337685	0.344516	0.35175	0.358079	0.36515	0.372607	0.379988
Malawi	0.21675	0.21909	0.220245	0.222084	0.223261	0.225432	0.228416	0.23211	0.235545	0.239258	0.243015	0.246257	0.250061	0.254371	0.259384	0.264873	0.271117	0.278919	0.287665	0.296451	0.305452	0.314531	0.322779	0.331074	0.339273	0.347076	0.354591	0.362256	0.370203	0.378644	0.38668	0.394762	0.402512	0.409533
Malaysia	0.56885	0.574411	0.580816	0.588913	0.597799	0.606701	0.616198	0.626721	0.636652	0.645848	0.654898	0.663047	0.670079	0.676296	0.68219	0.687714	0.692926	0.697664	0.702313	0.706868	0.711872	0.716779	0.722197	0.727267	0.732166	0.736876	0.741155	0.745785	0.750679	0.755794	0.760096	0.763675	0.767055	0.770371
Maldives	0.334014	0.346419	0.359806	0.373841	0.388632	0.403872	0.419671	0.435804	0.451534	0.46623	0.479842	0.491828	0.50295	0.513774	0.524094	0.532291	0.541772	0.551286	0.560687	0.568556	0.576606	0.585114	0.593311	0.601535	0.60986	0.617797	0.625293	0.632289	0.638795	0.644994	0.646877	0.650923	0.655723	0.660889
Mali	0.128314	0.130925	0.133365	0.13607	0.138916	0.141988	0.14554	0.149241	0.153062	0.157178	0.161125	0.165613	0.169898	0.174294	0.178301	0.182528	0.186953	0.191437	0.196188	0.20133	0.206701	0.212162	0.217406	0.222642	0.228294	0.234313	0.240649	0.247053	0.253581	0.260151	0.266357	0.272567	0.278852	0.284971
Malta	0.665997	0.670945	0.676519	0.682694	0.688813	0.693879	0.697978	0.703179	0.709563	0.716385	0.724045	0.730609	0.73614	0.74112	0.745611	0.749957	0.754042	0.758269	0.76252	0.76644	0.77087	0.775398	0.780207	0.784885	0.789647	0.794831	0.799698	0.804923	0.8101	0.814941	0.818077	0.822169	0.826498	0.830491
Marshall Islands	0.439759	0.444699	0.449857	0.455001	0.460315	0.466081	0.470011	0.472981	0.475691	0.478161	0.480771	0.484421	0.488362	0.491741	0.495176	0.499083	0.503401	0.508255	0.512034	0.51631	0.521171	0.525992	0.530793	0.536237	0.541626	0.547412	0.55363	0.560487	0.568203	0.577059	0.584907	0.592441	0.599193	0.605458
Mauritania	0.342049	0.347294	0.352687	0.358337	0.363785	0.369109	0.374479	0.378869	0.382793	0.386374	0.388944	0.390825	0.392541	0.394601	0.396962	0.400008	0.405082	0.40989	0.414677	0.419486	0.424951	0.430953	0.437128	0.443592	0.450105	0.456831	0.463767	0.471382	0.479315	0.487221	0.494846	0.502319	0.510257	0.518009
Mauritius	0.559592	0.565573	0.572324	0.57987	0.587482	0.594388	0.600522	0.606243	0.6113	0.615454	0.620235	0.624878	0.629235	0.633913	0.6393	0.64521	0.651077	0.656977	0.662865	0.668245	0.673386	0.679072	0.685715	0.692692	0.699225	0.704887	0.709666	0.714228	0.718749	0.723194	0.726156	0.729285	0.732685	0.736236
Mexico	0.516307	0.520854	0.526396	0.532459	0.538809	0.543969	0.549083	0.554459	0.560122	0.565931	0.57154	0.57693	0.582746	0.588943	0.594899	0.599714	0.603519	0.606636	0.609823	0.612003	0.614592	0.618753	0.624424	0.631024	0.638091	0.645467	0.653098	0.66064	0.667958	0.674492	0.679468	0.684255	0.688918	0.693437
Micronesia (Federated States of)	0.466923	0.473187	0.479376	0.485772	0.491226	0.496771	0.501268	0.504728	0.508374	0.511958	0.515967	0.51992	0.523873	0.527963	0.531521	0.535304	0.539013	0.542517	0.545596	0.548818	0.552379	0.556248	0.559754	0.56263	0.565181	0.568361	0.571628	0.575304	0.579009	0.582986	0.586529	0.590041	0.593438	0.59684
Monaco	0.847233	0.850359	0.8534	0.856317	0.859149	0.861959	0.864678	0.867318	0.870003	0.87255	0.87509	0.877602	0.880035	0.882446	0.884846	0.88724	0.889567	0.891864	0.894163	0.896408	0.898593	0.900768	0.902815	0.90483	0.906757	0.90876	0.910652	0.91248	0.914249	0.915977	0.91772	0.919435	0.921094	0.922701
Mongolia	0.474471	0.480874	0.486971	0.492827	0.499093	0.506305	0.513373	0.520692	0.527433	0.533993	0.5405	0.547129	0.553802	0.560383	0.567096	0.573216	0.579102	0.584819	0.589871	0.593283	0.596724	0.601143	0.605853	0.610521	0.615232	0.61942	0.623518	0.628287	0.633748	0.639953	0.64518	0.650109	0.654997	0.659698
Montenegro	0.665913	0.666051	0.664489	0.66063	0.657082	0.654848	0.655333	0.657079	0.660074	0.66262	0.667153	0.672652	0.678472	0.684456	0.690355	0.695887	0.701733	0.708284	0.715271	0.721446	0.728014	0.734592	0.740157	0.745484	0.750417	0.755259	0.759925	0.764564	0.769333	0.774193	0.776716	0.780487	0.784451	0.788566
Morocco	0.367752	0.375157	0.381821	0.387954	0.394616	0.400546	0.407275	0.413448	0.419872	0.426007	0.432003	0.438342	0.444771	0.451551	0.458515	0.465535	0.473083	0.480813	0.488979	0.497507	0.50613	0.515007	0.523739	0.532463	0.541	0.549677	0.557971	0.566341	0.574655	0.582887	0.590068	0.597791	0.605191	0.612385
Mozambique	0.178489	0.181267	0.183072	0.185404	0.187818	0.189774	0.192556	0.195799	0.199542	0.203608	0.207105	0.211279	0.215773	0.22032	0.2251	0.229922	0.23489	0.24014	0.245592	0.250917	0.256152	0.261315	0.266916	0.272863	0.279306	0.286629	0.293906	0.300942	0.307922	0.315002	0.321649	0.328289	0.335038	0.341791
Myanmar	0.325769	0.328462	0.331952	0.335892	0.340469	0.345685	0.35125	0.356934	0.362667	0.368987	0.376161	0.383898	0.392119	0.401051	0.410531	0.420319	0.430047	0.439427	0.447946	0.455906	0.463434	0.470286	0.475831	0.482321	0.488682	0.494766	0.500553	0.506286	0.512178	0.518005	0.522942	0.526047	0.529081	0.532025
Namibia	0.436798	0.441582	0.447243	0.453189	0.459595	0.465855	0.471571	0.476898	0.482221	0.487596	0.492987	0.49791	0.502668	0.507103	0.511927	0.516696	0.522241	0.528108	0.534224	0.540179	0.546875	0.554299	0.562214	0.570175	0.57821	0.585878	0.592423	0.597917	0.602735	0.606919	0.609895	0.612961	0.616323	0.619642
Nauru	0.561647	0.559345	0.556427	0.553	0.549751	0.546497	0.542976	0.539624	0.536036	0.532691	0.529757	0.527451	0.525238	0.523763	0.52257	0.521401	0.520376	0.519446	0.525625	0.530755	0.535556	0.54183	0.551268	0.5599	0.569157	0.576866	0.584453	0.590529	0.596479	0.603075	0.609367	0.615467	0.621209	0.626372
Nepal	0.208796	0.214736	0.22084	0.227258	0.234203	0.241108	0.248311	0.255785	0.263429	0.271316	0.279484	0.287838	0.29586	0.304044	0.312615	0.321382	0.330222	0.339103	0.348412	0.357845	0.367349	0.376384	0.384975	0.393498	0.402189	0.410781	0.418699	0.427226	0.435771	0.44424	0.451488	0.458389	0.46497	0.471007
Netherlands	0.804463	0.809393	0.814283	0.818742	0.823169	0.827518	0.830972	0.833971	0.837109	0.840111	0.843479	0.847306	0.850548	0.853598	0.856872	0.860073	0.863011	0.865837	0.868693	0.87105	0.87381	0.876958	0.879797	0.882335	0.884877	0.887519	0.89006	0.892792	0.895582	0.898548	0.900765	0.903288	0.905936	0.9084
New Zealand	0.753765	0.757306	0.761148	0.765255	0.768718	0.771606	0.775128	0.779077	0.782392	0.785132	0.78804	0.791872	0.796052	0.799941	0.803066	0.804586	0.804487	0.804611	0.80627	0.809489	0.813978	0.818805	0.823789	0.829123	0.833839	0.838721	0.843068	0.847329	0.851732	0.855957	0.859654	0.86217	0.864007	0.866431
Nicaragua	0.357042	0.362659	0.368567	0.374332	0.380308	0.386839	0.393876	0.401362	0.409081	0.416958	0.424555	0.431796	0.438394	0.444367	0.450008	0.45524	0.459936	0.464575	0.46908	0.472798	0.476563	0.480578	0.484817	0.489112	0.493725	0.498761	0.504064	0.509423	0.513996	0.518109	0.521892	0.526554	0.53113	0.535692
Niger	0.151828	0.153904	0.155862	0.157569	0.15904	0.160364	0.161531	0.162393	0.163444	0.164629	0.165989	0.168059	0.170772	0.173986	0.177653	0.182286	0.187277	0.192498	0.19774	0.203344	0.209833	0.216812	0.224518	0.232468	0.240782	0.249251	0.2578	0.266294	0.274734	0.283059	0.291119	0.298927	0.306941	0.314415
Nigeria	0.297794	0.301085	0.304523	0.307727	0.310672	0.313567	0.316752	0.319723	0.322783	0.326101	0.330048	0.334725	0.340808	0.347578	0.355111	0.363824	0.372533	0.380862	0.388408	0.395927	0.403976	0.412152	0.42045	0.428864	0.437654	0.446565	0.455235	0.463813	0.472343	0.480664	0.488581	0.496497	0.504462	0.511953
Niue	0.602137	0.607941	0.613405	0.618548	0.623286	0.627761	0.631974	0.636025	0.639653	0.643095	0.646667	0.650401	0.654398	0.65949	0.664148	0.670234	0.675765	0.681066	0.68587	0.689223	0.692548	0.69575	0.698661	0.70268	0.707109	0.711512	0.715979	0.72003	0.724651	0.729471	0.734311	0.73926	0.744077	0.74849
North Macedonia	0.606722	0.609485	0.61157	0.613011	0.614706	0.617353	0.621314	0.626195	0.631624	0.637449	0.644152	0.650639	0.657339	0.664971	0.672561	0.679136	0.684802	0.690184	0.69561	0.700456	0.705333	0.710095	0.714434	0.718719	0.722843	0.727088	0.731567	0.735906	0.740199	0.744403	0.747703	0.751492	0.755107	0.75848
Northern Mariana Islands	0.713248	0.717348	0.721392	0.725196	0.728942	0.732872	0.73688	0.740738	0.744483	0.747978	0.751139	0.753337	0.75442	0.755767	0.757488	0.758306	0.759031	0.75978	0.760096	0.759141	0.758893	0.758623	0.759084	0.760403	0.762597	0.765441	0.772026	0.781567	0.787318	0.791442	0.791863	0.792052	0.79183	0.791104
Norway	0.802822	0.806986	0.811452	0.81622	0.820575	0.824972	0.830113	0.835627	0.840327	0.844439	0.849594	0.854976	0.859302	0.862525	0.865468	0.867862	0.869881	0.871546	0.872374	0.874652	0.878241	0.882001	0.885629	0.889191	0.892531	0.895789	0.899413	0.902881	0.906069	0.909158	0.911724	0.913914	0.915555	0.916928
Oman	0.4249	0.439821	0.45629	0.474536	0.494656	0.516138	0.538238	0.560237	0.58077	0.599274	0.615786	0.630492	0.643329	0.654505	0.664846	0.674136	0.683228	0.6914	0.699323	0.706383	0.712849	0.718748	0.724499	0.729934	0.735071	0.739951	0.744821	0.749769	0.755067	0.76033	0.765463	0.770781	0.775971	0.781269
Pakistan	0.315463	0.320792	0.326237	0.331707	0.337316	0.343108	0.348979	0.35455	0.359964	0.365534	0.37108	0.3763	0.381262	0.386298	0.391783	0.397639	0.403747	0.409952	0.416124	0.422453	0.428907	0.435501	0.441952	0.448464	0.454866	0.461366	0.467841	0.474371	0.481095	0.487644	0.49387	0.500373	0.506795	0.512723
Palau	0.687396	0.691971	0.69629	0.698801	0.701607	0.705702	0.710482	0.714954	0.718804	0.72133	0.722431	0.723842	0.725337	0.726094	0.727028	0.728164	0.729523	0.731192	0.732153	0.73242	0.732724	0.733828	0.735384	0.736763	0.738824	0.741892	0.745143	0.747906	0.750836	0.753718	0.755625	0.755876	0.755851	0.755897
Palestine	0.417843	0.421944	0.427515	0.433222	0.439623	0.44576	0.450998	0.456938	0.46361	0.470351	0.475574	0.479425	0.48172	0.484966	0.48977	0.495628	0.50111	0.506871	0.513382	0.520968	0.529281	0.538656	0.548668	0.558254	0.567441	0.576755	0.586731	0.596127	0.60489	0.613165	0.619438	0.626043	0.632651	0.63798
Panama	0.547974	0.552794	0.557523	0.561947	0.563993	0.56502	0.566478	0.570204	0.575768	0.581347	0.5884	0.596152	0.602133	0.605847	0.608508	0.610886	0.61334	0.616109	0.619386	0.622472	0.624319	0.626173	0.630757	0.636386	0.641569	0.64739	0.653807	0.661564	0.672	0.684091	0.693767	0.702375	0.709256	0.715307
Papua New Guinea	0.333173	0.33791	0.342874	0.348668	0.354623	0.359579	0.364552	0.36841	0.371937	0.375329	0.378141	0.380511	0.382389	0.38441	0.386392	0.388757	0.391221	0.394086	0.396583	0.399651	0.403363	0.406534	0.409536	0.412464	0.416406	0.420487	0.424442	0.427998	0.430737	0.433395	0.435279	0.435592	0.436156	0.436714
Paraguay	0.474012	0.479053	0.484301	0.490054	0.496243	0.502865	0.50943	0.516218	0.522759	0.528914	0.534449	0.539757	0.545538	0.551474	0.557485	0.563351	0.569398	0.575845	0.582705	0.589128	0.596602	0.604102	0.610693	0.61762	0.624521	0.631015	0.637349	0.643597	0.649553	0.654924	0.659779	0.664794	0.66947	0.674089
Peru	0.51358	0.515145	0.516619	0.518685	0.522112	0.526593	0.531459	0.537081	0.542674	0.54822	0.553796	0.55912	0.564622	0.570126	0.575789	0.5818	0.588389	0.595522	0.603039	0.609887	0.617006	0.624103	0.631033	0.637725	0.643695	0.64915	0.654371	0.659483	0.664605	0.669604	0.673011	0.677448	0.681639	0.685608
Philippines	0.515982	0.519654	0.52328	0.527013	0.531126	0.535422	0.539646	0.543273	0.546054	0.548636	0.551174	0.553299	0.555237	0.556987	0.558661	0.560211	0.56199	0.564212	0.566957	0.570031	0.574283	0.579738	0.586593	0.594047	0.601998	0.61031	0.618963	0.627811	0.636552	0.645249	0.651748	0.657872	0.663856	0.669416
Poland	0.633094	0.6386	0.646779	0.655129	0.664287	0.673226	0.681181	0.689663	0.698116	0.70627	0.714843	0.723482	0.731245	0.737955	0.744225	0.749612	0.754351	0.758834	0.764143	0.770768	0.778216	0.785218	0.791163	0.796561	0.801451	0.8059	0.809732	0.814087	0.819615	0.82582	0.831686	0.837646	0.842781	0.847008
Portugal	0.616033	0.624516	0.632868	0.641044	0.649121	0.656199	0.662325	0.668468	0.67428	0.679743	0.685873	0.692155	0.69805	0.703641	0.708841	0.713858	0.718541	0.722737	0.726755	0.730737	0.735526	0.740646	0.745595	0.749912	0.753367	0.756357	0.759411	0.762949	0.766824	0.77139	0.775425	0.779179	0.782656	0.786464
Puerto Rico	0.684762	0.689449	0.693485	0.697485	0.702134	0.707046	0.711759	0.716757	0.722334	0.727737	0.734602	0.743679	0.75069	0.754752	0.757766	0.761183	0.765489	0.769869	0.773103	0.775904	0.779218	0.783377	0.789105	0.795798	0.802874	0.810947	0.819676	0.827411	0.833019	0.837466	0.840743	0.843196	0.845525	0.847509
Qatar	0.628092	0.637002	0.647552	0.658564	0.66982	0.680899	0.690782	0.701362	0.711246	0.719796	0.726666	0.733355	0.740444	0.747998	0.755909	0.763772	0.771016	0.77763	0.783577	0.789183	0.794427	0.799153	0.80372	0.808309	0.81131	0.8182	0.823553	0.829006	0.834551	0.840029	0.845319	0.850304	0.854918	0.85959
Republic of Korea	0.701221	0.711159	0.72055	0.73001	0.739482	0.748803	0.758012	0.766623	0.773912	0.78196	0.790187	0.797822	0.80494	0.811196	0.816835	0.821854	0.826598	0.831256	0.83576	0.839699	0.84382	0.84788	0.851785	0.855648	0.859463	0.863346	0.867378	0.871363	0.875314	0.879002	0.882216	0.885573	0.888634	0.891663
Republic of Moldova	0.594864	0.597642	0.599796	0.604235	0.608288	0.612584	0.615945	0.618349	0.618547	0.617142	0.615997	0.6165	0.618853	0.622403	0.627479	0.633378	0.639483	0.645163	0.651225	0.655854	0.661065	0.666601	0.671193	0.675943	0.680856	0.685917	0.692115	0.699094	0.706088	0.712935	0.717889	0.72444	0.729404	0.733719
Romania	0.608808	0.616489	0.620364	0.623786	0.628282	0.633483	0.637844	0.640926	0.6443	0.648518	0.654452	0.661333	0.66704	0.67125	0.67633	0.681495	0.687236	0.692499	0.698469	0.705118	0.711516	0.71537	0.717389	0.720256	0.724549	0.728433	0.7305	0.733751	0.740397	0.747294	0.75213	0.75724	0.763293	0.768757
Russian Federation	0.679892	0.688634	0.697135	0.70132	0.703326	0.707213	0.710967	0.713243	0.714744	0.716394	0.718122	0.719878	0.72261	0.727917	0.735381	0.742632	0.748875	0.754914	0.761092	0.766399	0.771621	0.775615	0.779858	0.784272	0.788194	0.792188	0.797442	0.802847	0.807503	0.811176	0.814037	0.817342	0.819904	0.822524
Rwanda	0.282729	0.283746	0.284869	0.284972	0.278627	0.275658	0.275621	0.27733	0.279967	0.283075	0.287685	0.293714	0.301554	0.309214	0.317132	0.324842	0.332605	0.340179	0.347864	0.355267	0.362262	0.369166	0.37607	0.38266	0.389702	0.397562	0.405643	0.413579	0.421754	0.430259	0.437425	0.445425	0.453753	0.462113
Saint Kitts and Nevis	0.580199	0.589771	0.598934	0.606782	0.61405	0.620288	0.625777	0.630988	0.635581	0.640032	0.645526	0.652652	0.662351	0.671948	0.680299	0.687116	0.692092	0.696195	0.701282	0.706364	0.712017	0.717691	0.722296	0.725882	0.728921	0.731572	0.734675	0.738084	0.741578	0.745049	0.746826	0.74884	0.751542	0.754464
Saint Lucia	0.509242	0.519668	0.529686	0.538969	0.547943	0.556952	0.565972	0.574379	0.582758	0.590595	0.597588	0.603677	0.609683	0.616195	0.622956	0.629202	0.635116	0.640991	0.64692	0.652236	0.657082	0.661841	0.666022	0.669834	0.673521	0.677144	0.680921	0.684894	0.689009	0.692891	0.693815	0.695816	0.699296	0.702888
Saint Vincent and the Grenadines	0.484276	0.488827	0.492819	0.497237	0.50219	0.508599	0.515107	0.521369	0.527345	0.533351	0.539495	0.545705	0.552038	0.558358	0.563928	0.56832	0.572461	0.576271	0.5801	0.584096	0.58777	0.590855	0.593871	0.597657	0.602411	0.608591	0.615865	0.623231	0.6306	0.637534	0.643515	0.648676	0.65351	0.658463
Samoa	0.493901	0.497321	0.500265	0.503212	0.505493	0.508324	0.511682	0.514905	0.517994	0.520907	0.524233	0.528101	0.532359	0.536827	0.541318	0.546239	0.550782	0.554947	0.559183	0.562835	0.566537	0.570193	0.572998	0.575508	0.577863	0.580636	0.584143	0.587507	0.590834	0.594629	0.598012	0.600357	0.602083	0.60449
San Marino	0.784627	0.790362	0.795493	0.800696	0.805851	0.811194	0.816325	0.821258	0.826245	0.831425	0.835637	0.838157	0.840589	0.842943	0.845207	0.847402	0.849555	0.85166	0.85378	0.85597	0.858231	0.86053	0.862823	0.865093	0.86714	[illegible]	0.86786	0.868788	0.870201	0.872187	0.873722	0.876536	0.879771	0.883012
Sao Tome and Principe	0.318917	0.319894	0.320663	0.321489	0.322534	0.32381	0.325628	0.327805	0.330639	0.334069	0.338034	0.342902	0.347966	0.353623	0.359519	0.365889	0.373177	0.380593	0.388696	0.397318	0.406143	0.415917	0.425517	0.435744	0.44615	0.456085	0.465762	0.475216	0.484341	0.492996	0.501247	0.509031	0.516252	0.522633
Saudi Arabia	0.443694	0.469297	0.494904	0.519642	0.543087	0.564776	0.584824	0.602719	0.618433	0.632116	0.644675	0.655908	0.666087	0.676489	0.687183	0.69813	0.708998	0.719365	0.729885	0.739033	0.748058	0.757717	0.766616	0.774456	0.781639	0.788038	0.793833	0.799196	0.804555	0.809585	0.81374	0.81833	0.823395	0.828451
Senegal	0.241283	0.248119	0.254365	0.259907	0.264599	0.268702	0.272559	0.27636	0.280364	0.284517	0.288163	0.291681	0.295119	0.298903	0.302915	0.306968	0.311043	0.315217	0.31982	0.325024	0.330559	0.336288	0.34257	0.349036	0.355494	0.361703	0.367606	0.37354	0.379762	0.386242	0.392492	0.399144	0.405958	0.412747
Serbia	0.631475	0.636911	0.63952	0.638966	0.637854	0.637144	0.638183	0.639807	0.64248	0.645911	0.649404	0.653494	0.658995	0.66491	0.672901	0.681552	0.689693	0.697346	0.705087	0.713425	0.723822	0.73407	0.740968	0.745823	0.749579	0.753083	0.756604	0.760297	0.764131	0.768124	0.77171	0.775814	0.780132	0.784525
Seychelles	0.595516	0.60208	0.608458	0.615607	0.623605	0.632149	0.64174	0.651046	0.658797	0.665658	0.671976	0.676755	0.680505	0.683111	0.685353	0.688853	0.692557	0.69534	0.695775	0.696289	0.698488	0.701697	0.705191	0.708914	0.712627	0.715573	0.719058	0.723769	0.72956	0.735536	0.739758	0.744277	0.748714	0.753017
Sierra Leone	0.215931	0.217984	0.218558	0.219444	0.22131	0.222622	0.222606	0.222014	0.221415	0.220105	0.220217	0.222422	0.227217	0.232497	0.238081	0.244272	0.251313	0.259124	0.267363	0.275704	0.283972	0.292125	0.301347	0.312424	0.322753	0.329746	0.336802	0.343657	0.350829	0.357865	0.364193	0.370616	0.376943	0.383229
Singapore	0.697561	0.706408	0.714699	0.723505	0.732324	0.741019	0.749978	0.758532	0.765799	0.770846	0.77761	0.784553	0.790121	0.794886	0.800467	0.807121	0.814123	0.821358	0.829027	0.834443	0.838312	0.841629	0.845157	0.848552	0.851321	0.854227	0.85693	0.859035	0.861113	0.863032	0.864906	0.866699	0.868313	0.869994
Slovakia	0.651682	0.656116	0.663259	0.673691	0.686294	0.695641	0.702674	0.709771	0.717092	0.724014	0.731743	0.738918	0.744094	0.748266	0.75264	0.758298	0.764065	0.769099	0.773273	0.776974	0.782214	0.787477	0.79149	0.793877	0.795676	0.796579	0.796887	0.798112	0.800024	0.802154	0.803524	0.806352	0.810059	0.813142
Slovenia	0.718435	0.723747	0.728512	0.732995	0.737532	0.742288	0.747644	0.75356	0.759032	0.764369	0.770695	0.777403	0.78296	0.787467	0.792062	0.796736	0.800885	0.804228	0.807682	0.810407	0.812884	0.814983	0.816458	0.818196	0.820305	0.822518	0.824169	0.82599	0.828677	0.831949	0.834833	0.838414	0.841727	0.844866
Solomon Islands	0.318675	0.323284	0.328902	0.334608	0.34079	0.347561	0.353929	0.359558	0.364887	0.369731	0.372795	0.374738	0.376133	0.37776	0.379901	0.382641	0.385955	0.389777	0.394353	0.399113	0.404756	0.410817	0.416416	0.421881	0.426785	0.431258	0.435918	0.440402	0.444577	0.448369	0.451188	0.453728	0.455823	0.458023
Somalia	0.047951	0.048428	0.048871	0.05663	0.050105	0.05082	0.051493	0.05217	0.052879	0.053605	0.054366	0.055148	0.05596	0.056771	0.057588	0.058402	0.074884	0.088521	0.099892	0.110145	0.119659	0.129	0.138091	0.143735	0.152004	0.1643	0.177547	0.189597	0.199399	0.207782	0.214009	0.219701	0.224686	0.229019
South Africa	0.546236	0.552326	0.558049	0.563586	0.569043	0.574667	0.580183	0.585493	0.59046	0.595304	0.600077	0.604106	0.607659	0.610975	0.614387	0.618179	0.622738	0.628045	0.633458	0.63841	0.64348	0.648759	0.654216	0.659783	0.665225	0.670622	0.675796	0.680663	0.685169	0.689367	0.69264	0.696174	0.699586	0.702613
South Sudan	0.278221	0.280256	0.282098	0.283814	0.285549	0.287483	0.289637	0.292046	0.294622	0.297471	0.300693	0.304187	0.307988	0.312194	0.316867	0.321977	0.327468	0.333489	0.339767	0.346452	0.353183	0.359632	0.361824	0.365129	0.368035	0.370173	0.370757	0.369714	0.368645	0.369677	0.371257	0.373985	0.37706	0.379081
Spain	0.669202	0.677131	0.684972	0.692355	0.699324	0.705737	0.711302	0.716653	0.721751	0.726468	0.731241	0.735837	0.740386	0.744738	0.748936	0.752773	0.755996	0.759151	0.763337	0.767925	0.772315	0.776054	0.779118	0.7816	0.783728	0.786288	0.789049	0.792289	0.795707	0.799402	0.802322	0.80541	0.80858	0.81182
Sri Lanka	0.543049	0.548435	0.553366	0.558917	0.565019	0.571281	0.57796	0.584654	0.590289	0.594876	0.59953	0.603257	0.607425	0.612306	0.617546	0.622984	0.628759	0.634773	0.640931	0.647096	0.653597	0.660728	0.668537	0.676201	0.683886	0.691177	0.69809	0.70471	0.710863	0.716521	0.721062	0.725546	0.728566	0.730698
Sudan	0.303373	0.308627	0.314018	0.31941	0.324883	0.33071	0.336622	0.343353	0.350552	0.357999	0.365675	0.373723	0.382018	0.390608	0.399432	0.40895	0.41939	0.430134	0.440951	0.451729	0.46298	0.474791	0.484151	0.4935	0.503325	0.512996	0.522674	0.532112	0.540932	0.549015	0.556095	0.56282	0.569088	0.573101
Suriname	0.521404	0.526057	0.530023	0.532938	0.535335	0.53788	0.5416	0.546265	0.551171	0.555557	0.560617	0.566061	0.571841	0.577971	0.583984	0.589427	0.594301	0.598882	0.603741	0.608608	0.613717	0.618853	0.623706	0.628531	0.632981	0.636832	0.639681	0.642517	0.645367	0.648088	0.649482	0.651023	0.652845	0.65487
Sweden	0.797042	0.801775	0.807297	0.812974	0.818761	0.824555	0.829709	0.83434	0.838573	0.842403	0.846045	0.849125	0.852038	0.85503	0.857933	0.860346	0.862685	0.865005	0.867339	0.869331	0.872324	0.875645	0.878508	0.881333	0.884056	0.886736	0.889217	0.891847	0.894813	0.898331	0.901469	0.904935	0.908267	0.910928
Switzerland	0.873003	0.875097	0.878169	0.881427	0.883801	0.885571	0.887463	0.889373	0.890977	0.89295	0.896206	0.899484	0.901896	0.903776	0.905876	0.908254	0.910909	0.913316	0.916705	0.919022	0.921544	0.924359	0.926902	0.929137	0.931191	0.933095	0.934752	0.936554	0.938414	0.940292	0.942014	0.943587	0.944908	0.946051
Syrian Arab Republic	0.417624	0.422509	0.42765	0.432659	0.437435	0.441956	0.446164	0.449843	0.453551	0.456656	0.459816	0.463294	0.467515	0.472033	0.478152	0.486437	0.494532	0.503112	0.511698	0.520018	0.528704	0.536926	0.542412	0.544706	0.545346	0.546193	0.548511	0.552215	0.557159	0.563632	0.570155	0.576828	0.583282	0.588682
Taiwan (Province of China)	0.677908	0.686733	0.694695	0.702461	0.71031	0.718497	0.727754	0.737736	0.746499	0.752981	0.759838	0.767278	0.774933	0.782236	0.789317	0.796286	0.803257	0.810655	0.81753	0.82319	0.828471	0.832664	0.836848	0.841108	0.845017	0.848845	0.852722	0.856648	0.860382	0.86418	0.868363	0.873013	0.877455	0.881538
Tajikistan	0.478794	0.483649	0.484887	0.484782	0.482638	0.479105	0.473823	0.468266	0.462543	0.457708	0.454905	0.455071	0.458822	0.464464	0.471257	0.477391	0.482764	0.487453	0.491269	0.494425	0.497658	0.500933	0.504544	0.507739	0.511506	0.514611	0.518038	0.522361	0.526597	0.531184	0.535658	0.540551	0.545706	0.551182
Thailand	0.499393	0.508252	0.517278	0.526283	0.53514	0.544112	0.552726	0.559773	0.565071	0.569918	0.57424	0.578126	0.582047	0.586191	0.590513	0.594838	0.599386	0.604279	0.609785	0.615756	0.621066	0.624934	0.629909	0.635595	0.641266	0.647012	0.652881	0.658825	0.664802	0.670528	0.675101	0.679413	0.683479	0.687188
Timor-Leste	0.275587	0.28385	0.292707	0.302046	0.31152	0.320702	0.33001	0.338723	0.346379	0.349925	0.353946	0.359413	0.364065	0.368423	0.372674	0.376941	0.380655	0.385311	0.391739	0.398176	0.405463	0.412886	0.420201	0.427006	0.433587	0.439476	0.445048	0.449345	0.452871	0.458626	0.465876	0.476931	0.483069	0.484533
Togo	0.289819	0.294925	0.299414	0.302289	0.306107	0.310325	0.314603	0.31911	0.322422	0.325627	0.328479	0.331227	0.334261	0.338033	0.341429	0.344295	0.347436	0.350802	0.354778	0.359139	0.363952	0.369406	0.375706	0.382524	0.389731	0.397487	0.405711	0.413767	0.421795	0.429846	0.437427	0.445182	0.453014	0.460522
Tokelau	0.55541	0.559987	0.564366	0.568601	0.572797	0.577219	0.581879	0.586914	0.592197	0.597702	0.603563	0.609475	0.615233	0.62084	0.626583	0.632508	0.638772	0.645637	0.652616	0.6591	0.665841	0.672595	0.679178	0.687199	0.693492	0.701191	0.708407	0.715386	0.722154	0.72871	0.733957	0.739372	0.744719	0.749949
Tonga	0.512413	0.517796	0.522106	0.525952	0.529704	0.534201	0.538442	0.542245	0.546158	0.550264	0.554333	0.558251	0.56235	0.566184	0.569259	0.571917	0.574183	0.576157	0.578674	0.580482	0.582447	0.585369	0.588464	0.591909	0.595969	0.600377	0.605857	0.611634	0.616915	0.621652	0.625975	0.629558	0.632705	0.635995
Trinidad and Tobago	0.614066	0.618623	0.623627	0.628558	0.633444	0.638376	0.643453	0.648741	0.654214	0.660347	0.666902	0.673059	0.679199	0.6862	0.69316	0.699539	0.706404	0.712956	0.719464	0.72448	0.729757	0.734916	0.739914	0.745004	0.750079	0.754666	0.758216	0.760909	0.763053	0.764846	0.765903	0.767133	0.768677	0.770449
Tunisia	0.471402	0.479671	0.488346	0.496908	0.505524	0.514126	0.523213	0.531988	0.540421	0.548506	0.556249	0.563833	0.570957	0.57791	0.584812	0.591427	0.597993	0.604676	0.611182	0.617471	0.623533	0.628859	0.634054	0.638999	0.643828	0.648451	0.652961	0.657473	0.662081	0.666784	0.670473	0.674816	0.679335	0.683722
Turkey	0.478246	0.48627	0.49427	0.502576	0.509862	0.517358	0.525058	0.532953	0.54074	0.547733	0.554995	0.561407	0.567785	0.574111	0.580993	0.588307	0.595994	0.603613	0.610916	0.617298	0.624069	0.631327	0.63849	0.646118	0.654097	0.662687	0.67119	0.679948	0.688717	0.697075	0.705101	0.713858	0.722501	0.730952

Turkmenistan	0.576123	0.577094	0.577064	0.578109	0.577919	0.57775	0.577483	0.575993	0.574763	0.575056	0.577871	0.581761	0.586492	0.591678	0.5976	0.604028	0.609966	0.615424	0.620594	0.625626	0.630795	0.6364	0.641573	0.646381	0.651107	0.655697	0.659928	0.663933	0.667833	0.67129	0.674065	0.67667	0.679337	0.682175
Tuvalu	0.445878	0.45449	0.462491	0.469987	0.477826	0.483852	0.488215	0.493135	0.499593	0.505373	0.510901	0.516153	0.522298	0.527434	0.531901	0.535606	0.539142	0.543081	0.547445	0.550632	0.55334	0.556758	0.559442	0.562649	0.565966	0.570551	0.575481	0.580638	0.585527	0.592049	0.597233	0.60214	0.606665	0.611234
Uganda	0.184004	0.185362	0.186801	0.188731	0.191877	0.195958	0.201037	0.206999	0.213844	0.221478	0.229454	0.237944	0.2469	0.256004	0.265408	0.275615	0.286072	0.296686	0.307534	0.318325	0.328799	0.33887	0.347998	0.356857	0.365692	0.374744	0.383314	0.391842	0.400372	0.409184	0.417338	0.425655	0.434097	0.442281
Ukraine	0.652982	0.657425	0.663049	0.668634	0.671814	0.67366	0.674668	0.675549	0.676432	0.676463	0.67657	0.677607	0.679219	0.683016	0.689465	0.69575	0.701639	0.707486	0.713885	0.718935	0.723465	0.726945	0.730529	0.73547	0.740628	0.744286	0.747478	0.751193	0.755147	0.758915	0.761342	0.763815	0.763603	0.764531
United Arab Emirates	0.646232	0.663621	0.679505	0.693122	0.705458	0.717619	0.729334	0.740184	0.75022	0.759283	0.767155	0.77397	0.780223	0.786437	0.79314	0.800417	0.809296	0.81864	0.826664	0.832273	0.835136	0.836407	0.836539	0.835994	0.835337	0.835226	0.836632	0.83991	0.844234	0.848748	0.852775	0.856211	0.859201	0.86221
United Kingdom	0.764425	0.76924	0.774479	0.779926	0.784606	0.787673	0.790287	0.793597	0.797439	0.802279	0.807299	0.811355	0.814094	0.816179	0.818678	0.820959	0.82299	0.825207	0.827727	0.830373	0.83357	0.837226	0.842151	0.84725	0.851268	0.854835	0.858553	0.862535	0.866419	0.870414	0.873099	0.875767	0.878242	0.880445
United Republic of Tanzania	0.262941	0.26575	0.268049	0.26987	0.271599	0.273926	0.277201	0.28087	0.284717	0.288983	0.293513	0.298434	0.303801	0.309518	0.315783	0.32255	0.329405	0.336412	0.343412	0.350578	0.358014	0.36557	0.372606	0.379791	0.387188	0.394965	0.403095	0.411343	0.419875	0.428852	0.438059	0.447547	0.456953	0.465889
United States Virgin Islands	0.662472	0.667347	0.670906	0.674974	0.681607	0.690542	0.69965	0.707028	0.712172	0.715905	0.719159	0.723767	0.734048	0.74219	0.748866	0.754903	0.760529	0.765608	0.76973	0.772116	0.775054	0.779528	0.785154	0.791956	0.798373	0.802715	0.804126	0.80362	0.80371	0.805657	0.807987	0.810753	0.813837	0.816423
United States of America	0.773328	0.77572	0.779206	0.782549	0.785597	0.788622	0.791098	0.792922	0.794725	0.797166	0.800877	0.805354	0.809157	0.811825	0.813729	0.814288	0.81491	0.817765	0.822611	0.828379	0.833914	0.838045	0.841609	0.844912	0.848174	0.851769	0.855607	0.859209	0.862611	0.866052	0.868921	0.87162	0.874146	0.876475
Uruguay	0.5951	0.599158	0.602921	0.605487	0.607892	0.609249	0.612282	0.618414	0.625767	0.632171	0.636325	0.63887	0.641518	0.644697	0.647665	0.650442	0.654127	0.660261	0.668342	0.675744	0.681531	0.682264	0.680305	0.681906	0.686791	0.694033	0.703347	0.712794	0.720947	0.727658	0.732703	0.737691	0.742056	0.745444
Uzbekistan	0.509085	0.510641	0.511954	0.515164	0.519096	0.524512	0.531222	0.538469	0.546008	0.553599	0.560522	0.56748	0.574224	0.580476	0.586439	0.591943	0.597003	0.602053	0.606848	0.611549	0.616134	0.620354	0.623916	0.62679	0.629193	0.63122	0.632229	0.632186	0.631417	0.630345	0.628973	0.628654	0.62977	0.632091
Vanuatu	0.358817	0.362394	0.365952	0.369259	0.373125	0.376928	0.38068	0.384721	0.388464	0.391846	0.39544	0.398321	0.400457	0.402811	0.405427	0.408504	0.412342	0.416295	0.42075	0.4253	0.429656	0.434125	0.438327	0.442015	0.445699	0.449189	0.452872	0.456983	0.461093	0.465345	0.468487	0.471253	0.47398	0.476543
Venezuela (Bolivarian Republic of)	0.523652	0.529321	0.536646	0.543689	0.549822	0.55527	0.558594	0.562578	0.566766	0.568982	0.571715	0.573784	0.570807	0.563168	0.558606	0.561965	0.571774	0.584489	0.595868	0.603103	0.609258	0.615052	0.62076	0.625239	0.626647	0.626316	0.627139	0.628504	0.625736	0.618607	0.609487	0.599865	0.590158	0.580213
Viet Nam	0.414175	0.419552	0.42612	0.433615	0.441851	0.450721	0.459964	0.470112	0.480374	0.489574	0.497644	0.505067	0.512845	0.521126	0.529461	0.537442	0.544835	0.551896	0.558568	0.564599	0.570333	0.575735	0.581146	0.586405	0.591766	0.597259	0.6027	0.608381	0.614297	0.620332	0.626096	0.631534	0.637309	0.643028
Yemen	0.210289	0.218545	0.227033	0.235566	0.244388	0.253415	0.262562	0.271859	0.281267	0.290685	0.300179	0.309648	0.319025	0.328494	0.338229	0.348749	0.358979	0.368887	0.378528	0.387813	0.397618	0.405674	0.413161	0.42042	0.427267	0.431131	0.433894	0.435604	0.437383	0.439206	0.440417	0.442042	0.444158	0.446394
Zambia	0.307658	0.309189	0.310339	0.311493	0.311676	0.312231	0.313333	0.314783	0.316172	0.318128	0.320809	0.324081	0.32784	0.332367	0.337637	0.343504	0.350342	0.358534	0.36734	0.376788	0.386884	0.397037	0.406906	0.41671	0.426454	0.435802	0.445063	0.454201	0.463238	0.472092	0.480289	0.488804	0.497381	0.505572
Zimbabwe	0.412723	0.420668	0.426362	0.431356	0.436516	0.44083	0.445831	0.449656	0.452672	0.455055	0.456665	0.458527	0.459639	0.458573	0.456081	0.451807	0.446091	0.440432	0.43336	0.429202	0.428181	0.43034	0.436635	0.444127	0.451356	0.458147	0.464284	0.470263	0.475682	0.479218	0.481203	0.484034	0.48746	0.491185
