# Supplementary material for: Trends, inequalities, and 2030 projections of women's tracheal, bronchial, and lung cancer attributable to air pollution, radon, and tobacco exposure
Source: Front Glob Womens Health. 2026 Aug 7;7:1662451. doi: 10.3389/fgwh.2026.1662451 (PMC13493495; doi:10.3389/fgwh.2026.1662451)
Supplement: Supplementary file 2 [file Table1.docx]

**The Process of Searching Data through the Online GBD Tool**

To accurately extract the target data, "Risk factor" should be selected in the "GBD Estimate" column; "Deaths" and "DALYs" should be selected in the "Measure" column; "Number" and "Rate" should be selected in the "Metric" column; "Ambient particulate matter pollution", "Household air pollution from solid fuels", "Residential radon", "Smoking", "Secondhand smoke" should be selected in the "Risk" column; "Tracheal, bronchus, and lung " should be selected in the "Cause" column; "Global", "Low SDI", "Low-middle SDI", "Middle SDI", "High-middle SDI", "High SDI", "Central Asia", "Central Europe", "Eastern Europe", "Australasia", "High-income Asia Pacific", "High-income North America", "Southern Latin America", "Western Europe", "Andean Latin America", "Caribbean", "Central Latin America", "Tropical Latin America", "North Africa and Middle East", "South Asia", "East Asia", "Oceania", "Southeast Asia", "Central Sub-Saharan Africa", "Eastern Sub-Saharan Africa", "Southern Sub-Saharan Africa", "Western Sub-Saharan Africa" should be selected in the "Location" column; "30 to 34", "35 to 39", "40 to 44", "45 to 49", "50 to 54", "55 to 59", "60 to 64", "65 to 69", "70 to 74", "75 to 79", "80 to 84", "85 to 89", "90 to 94", and "95 plus" should be selected in the "Age" column; only "Female" should be selected in the "Sex" column; and all the years from 1990 to 2023 should be selected in the "Year" column.
