# Supplementary material for: Trends, inequalities, and 2030 projections of women's tracheal, bronchial, and lung cancer attributable to air pollution, radon, and tobacco exposure
Source: Front Glob Womens Health. 2026 Aug 7;7:1662451. doi: 10.3389/fgwh.2026.1662451 (PMC13493495; doi:10.3389/fgwh.2026.1662451)
Supplement: Supplementary file 3 [file Table2.docx]

| **The four common sequelae for cancer and corresponding DW in the GBD 2023 Study** | | | |
| --- | --- | --- | --- |
| **Sequelae** | **Health status** | **Lay description** | **DW (95% CI)** |
| Diagnosis/First therapy phase | With diagnosis, and primary therapy for cancer | Has pain, nausea, fatigue, weight loss and high anxiety. | 0.288 (0.193, 0.399) |
| Controlled phase | No generic uncomplicated disease for cancer, also with worry and daily medication | Has a chronic disease that requires medication every day and causes some worry but minimal interference with daily activities. | 0.049 (0.031, 0.072) |
| Metastatic phase | With metastatic occurrence | Has severe pain, extreme fatigue, weight loss and high anxiety. | 0.451 (0.307, 0.600) |
| Terminal phase | With medication for cancer terminal | Has lost a lot of weight and regularly uses strong medication to avoid constant pain, also has no appetite, feels nauseous, and needs to spend most of the day in bed. | 0.540 (0.377, 0.687) |
| **GBD: Global Burden of Disease**  **DW: Disability weight** | | | |
