# Supplementary material for: Trends, inequalities, and 2030 projections of women's tracheal, bronchial, and lung cancer attributable to air pollution, radon, and tobacco exposure
Source: Front Glob Womens Health. 2026 Aug 7;7:1662451. doi: 10.3389/fgwh.2026.1662451 (PMC13493495; doi:10.3389/fgwh.2026.1662451)
Supplement: Supplementary file 4 [file Table3.docx]

**R code for the recalculation of age-standardized rates (ASR) and their 95% uncertainty intervals (UI)**

library(tidyverse)

\# Data preparation

mydata <- mydata %>%

mutate(

Rate	= Number / Population * 100000,

Rate_U = Upper / Population * 100000,

Rate_L = Lower / Population * 100000,

SE	= (Rate_U - Rate_L) / (2 * 1.96)

)

\# Deterministic ASR (POINT ESTIMATE)

weight <- mydata$Weight / 100

ASR_estimate <- sum(mydata$Rate * weight)

\# Monte Carlo simulation (UNCERTAINTY ONLY)

set.seed(1234)

n_sim <- 100000

sim_matrix <- matrix(NA, nrow = nrow(mydata), ncol = n_sim)

for (i in 1:nrow(mydata)) {

sim_matrix[i, ] <- mydata$Rate[i] +

mydata$SE[i] * rnorm(n_sim)

}

ASR_sim <- colSums(sim_matrix * weight)

\# Final results

ASR_results <- data.frame(

estimate = ASR_estimate,

lower	= quantile(ASR_sim, 0.025),

upper	= quantile(ASR_sim, 0.975)

)
