# Supplementary material for: Trends, inequalities, and 2030 projections of women's tracheal, bronchial, and lung cancer attributable to air pollution, radon, and tobacco exposure
Source: Front Glob Womens Health. 2026 Aug 7;7:1662451. doi: 10.3389/fgwh.2026.1662451 (PMC13493495; doi:10.3389/fgwh.2026.1662451)
Supplement: Supplementary file 5 [file Table4.docx]

| **Table S1 ASDRs (per 10^5^) of female TBL cancer attributable to air pollution and tobacco exposure in 2023 with AAPCs from 1990 to 2023** | | | | | | | | | | |
| --- | --- | --- | --- | --- | --- | --- | --- | --- | --- | --- |
| Location | Ambient particulate matter pollution | | Household air pollution from solid fuels | | Residential radon | | Smoking | | Secondhand smoke | |
|  | ASDR in 2023 | AAPC (1990-2023) | ASDR in 2023 | AAPC (1990-2023) | ASDR in 2023 | AAPC (1990-2023) | ASDR in 2023 | AAPC (1990-2023) | ASDR in 2023 | AAPC (1990-2023) |
| Total Females | 85.280 (73.743, 96.898) | 0.450 (0.409, 0.482) | 27.303 (21.109, 33.496) | -4.114 (-4.196, -4.036) | 26.274 (13.507, 39.049) | -0.498 (-0.522, -0.475) | 199.986 (183.224, 216.741) | -1.157 (-1.193, -1.122) | 67.783 (62.064, 73.527) | -1.040 (-1.123, -0.959) |
| SDI Region |  |  |  |  |  |  |  |  |  |  |
| Low SDI | 21.295 (17.585, 24.986) | 3.044 (2.993, 3.092) | 75.370 (63.521, 87.201) | 0.832 (0.784, 0.881) | 10.545 (5.297, 15.782) | 1.535 (1.484, 1.581) | 29.692 (23.952, 35.368) | 0.737 (0.697, 0.778) | 27.604 (23.997, 31.205) | 1.237 (1.157, 1.331) |
| Low-middle SDI | 38.111 (32.312, 43.914) | 2.902 (2.853, 2.956) | 77.071 (64.798, 89.305) | 0.118 (0.056, 0.180) | 15.039 (7.603, 22.468) | 1.401 (1.337, 1.474) | 52.292 (44.267, 60.388) | 0.013 (-0.044, 0.067)* | 39.718 (35.163, 44.264) | 0.651 (0.573, 0.715) |
| Middle SDI | 75.331 (64.930, 85.667) | 3.174 (3.120, 3.225) | 30.973 (22.836, 39.083) | -3.885 (-3.977, -3.804) | 15.346 (7.987, 22.616) | 0.246 (0.182, 0.307) | 71.815 (62.388, 81.146) | -0.473 (-0.519, -0.424) | 80.450 (72.711, 88.245) | 0.469 (0.416, 0.518) |
| High-middle SDI | 116.197 (99.783, 132.513) | 1.690 (1.626, 1.756) | 19.766 (10.925, 28.527) | -6.773 (-6.939, -6.660) | 21.080 (10.803, 31.352) | -0.926 (-0.972, -0.879) | 108.079 (88.046, 128.064) | -2.055 (-2.233, -1.929) | 78.653 (70.602, 86.641) | -1.881 (-2.001, -1.762) |
| High SDI | 98.180 (84.353, 112.138) | -0.246 (-0.296, -0.206) | 5.935 (2.298, 9.559) | -6.896 (-7.151, -6.732) | 37.253 (18.324, 56.053) | -0.470 (-0.509, -0.435) | 347.906 (323.196, 372.714) | -0.825 (-0.868, -0.785) | 76.775 (70.126, 83.408) | -1.171 (-1.212, -1.130) |
| GBD Region |  |  |  |  |  |  |  |  |  |  |
| Central Asia | 40.442 (35.554, 45.357) | -1.315 (-1.427, -1.195) | 10.508 (8.265, 12.746) | -5.148 (-5.287, -5.005) | 18.256 (8.853, 27.712) | -2.356 (-2.462, -2.243) | 21.651 (17.467, 25.850) | -2.839 (-2.983, -2.628) | 31.760 (29.233, 34.291) | -2.359 (-2.465, -2.249) |
| Central Europe | 103.601 (88.922, 118.370) | -0.015 (-0.103, 0.073)* | 8.050 (3.476, 12.641) | -5.469 (-5.723, -5.288) | 55.154 (24.921, 85.093) | 1.131 (1.090, 1.167) | 550.661 (518.033, 583.492) | 1.144 (1.099, 1.180) | 81.734 (74.935, 88.507) | 0.027 (-0.016, 0.065)* |
| Eastern Europe | 23.253 (19.829, 26.672) | -3.997 (-4.105, -3.878) | 0.877 (0.496, 1.256) | -6.579 (-6.840, -6.299) | 22.384 (11.389, 33.351) | -1.092 (-1.156, -1.014) | 86.991 (75.915, 98.121) | 0.419 (0.327, 0.516) | 30.921 (28.295, 33.546) | -2.117 (-2.169, -2.054) |
| Australasia | 24.827 (19.508, 30.138) | -1.024 (-1.584, -0.420) | 0.365 (-0.232, 0.961) | -4.593 (-4.858, -4.436) | 12.603 (4.849, 20.350) | -0.471 (-0.545, -0.400) | 418.345 (380.055, 456.742) | -1.125 (-1.253, -1.036) | 34.003 (31.145, 36.875) | -2.504 (-2.640, -2.418) |
| High-income Asia Pacific | 45.635 (39.288, 52.029) | -0.608 (-0.712, -0.488) | 0.230 (-0.064, 0.529) | -6.325 (-6.526, -6.195) | 11.837 (6.039, 17.690) | -0.367 (-0.417, -0.333) | 152.088 (131.715, 172.228) | -1.430 (-1.513, -1.377) | 37.707 (34.442, 40.986) | -2.839 (-2.944, -2.756) |
| High-income North America | 31.923 (24.809, 39.059) | -4.956 (-5.093, -4.819) | 0.210 (-0.122, 0.539) | -1.797 (-1.867, -1.726) | 45.314 (20.697, 69.760) | -1.529 (-1.564, -1.488) | 614.483 (569.056, 659.452) | -1.892 (-1.947, -1.847) | 43.252 (39.537, 46.951) | -3.285 (-3.321, -3.251) |
| Southern Latin America | 76.471 (66.112, 86.857) | 1.039 (0.832, 1.249) | 1.074 (0.082, 2.070) | -8.315 (-8.692, -8.115) | 18.241 (6.318, 30.151) | 0.840 (0.733, 0.928) | 286.585 (254.654, 318.488) | 0.546 (0.369, 0.687) | 54.243 (49.754, 58.735) | -0.197 (-0.284, -0.102) |
| Western Europe | 47.600 (39.002, 56.178) | -2.166 (-2.354, -1.977) | 0.544 (-0.357, 1.445) | -2.689 (-2.783, -2.603) | 53.145 (25.048, 81.232) | 0.836 (0.795, 0.870) | 514.940 (488.052, 542.082) | 0.437 (0.390, 0.474) | 48.221 (44.459, 52.032) | -0.713 (-0.754, -0.681) |
| Andean Latin America | 67.727 (57.764, 77.595) | 0.415 (0.213, 0.661) | 14.316 (9.735, 18.890) | -4.287 (-4.606, -4.033) | 17.805 (6.913, 28.697) | 0.515 (0.394, 0.643) | 39.738 (30.472, 49.079) | -0.460 (-0.608, -0.313) | 20.966 (18.851, 23.078) | -0.818 (-0.954, -0.685) |
| Caribbean | 41.747 (34.910, 48.565) | 1.170 (0.993, 1.421) | 24.736 (19.926, 29.599) | -2.102 (-2.182, -2.029) | 13.452 (5.626, 21.234) | 0.401 (0.329, 0.472) | 265.429 (228.659, 302.247) | -0.115 (-0.276, 0.080)* | 57.547 (51.748, 63.366) | -0.153 (-0.249, -0.060) |
| Central Latin America | 39.673 (34.645, 44.723) | -1.595 (-1.662, -1.525) | 8.991 (7.181, 10.791) | -4.661 (-4.771, -4.578) | 16.426 (8.540, 24.337) | -1.282 (-1.335, -1.226) | 62.243 (52.969, 71.481) | -2.916 (-2.976, -2.850) | 22.291 (20.383, 24.204) | -2.808 (-2.874, -2.731) |
| Tropical Latin America | 52.364 (44.932, 59.774) | 0.774 (0.670, 0.897) | 3.460 (1.760, 5.165) | -6.258 (-6.550, -6.063) | 24.165 (10.333, 37.890) | 0.848 (0.789, 0.895) | 249.573 (223.765, 275.471) | -0.126 (-0.204, -0.063) | 36.347 (32.915, 39.743) | -1.428 (-1.469, -1.389) |
| North Africa and Middle East | 82.194 (71.685, 92.737) | 2.107 (2.004, 2.204) | 7.056 (5.690, 8.435) | -4.042 (-4.096, -3.981) | 13.958 (7.431, 20.462) | 0.779 (0.745, 0.820) | 77.482 (64.790, 90.239) | 0.451 (0.384, 0.511) | 49.925 (45.279, 54.589) | 0.770 (0.734, 0.810) |
| South Asia | 42.390 (34.514, 50.266) | 4.499 (4.374, 4.602) | 52.798 (43.631, 62.079) | -0.134 (-0.226, -0.055) | 12.154 (5.941, 18.336) | 1.864 (1.765, 1.954) | 31.788 (24.673, 38.897) | 0.588 (0.516, 0.675) | 31.306 (27.082, 35.561) | 0.923 (0.819, 1.018) |
| East Asia | 187.615 (158.398, 216.838) | 2.386 (2.265, 2.507) | 31.800 (18.147, 45.400) | -6.856 (-7.008, -6.739) | 33.666 (16.438, 51.043) | -0.855 (-0.903, -0.808) | 202.303 (153.313, 251.389) | -1.520 (-1.631, -1.443) | 137.518 (122.213, 152.837) | -1.399 (-1.525, -1.274) |
| Oceania | 23.431 (18.716, 28.123) | 1.777 (1.574, 2.074) | 293.595 (235.349, 352.144) | 0.962 (0.839, 1.071) | 31.166 (6.456, 55.714) | 1.286 (1.164, 1.388) | 284.044 (220.758, 347.205) | 0.825 (0.723, 0.913) | 153.653 (129.195, 178.337) | 1.451 (1.336, 1.546) |
| Southeast Asia | 78.697 (66.744, 90.622) | 2.224 (2.091, 2.395) | 47.880 (37.830, 57.862) | -2.964 (-3.095, -2.851) | 11.951 (5.976, 17.954) | 0.790 (0.712, 0.880) | 71.524 (58.718, 84.272) | -1.498 (-1.578, -1.407) | 91.688 (81.464, 101.904) | 0.419 (0.323, 0.531) |
| Central Sub-Saharan Africa | 21.440 (17.378, 25.502) | 2.448 (2.346, 2.548) | 98.947 (78.562, 119.428) | 1.188 (1.075, 1.287) | 11.853 (3.033, 20.708) | 1.579 (1.469, 1.675) | 12.823 (8.932, 16.721) | 1.307 (1.224, 1.400) | 20.864 (17.160, 24.555) | 1.449 (1.346, 1.544) |
| Eastern Sub-Saharan Africa | 7.251 (6.127, 8.375) | 2.617 (2.515, 2.721) | 62.590 (53.489, 71.596) | 1.096 (1.044, 1.159) | 6.923 (3.428, 10.427) | 1.427 (1.348, 1.492) | 11.305 (8.981, 13.613) | 0.818 (0.690, 0.939) | 10.323 (9.042, 11.597) | 0.427 (0.385, 0.471) |
| Southern Sub-Saharan Africa | 57.721 (49.108, 66.335) | 0.620 (0.477, 0.765) | 21.961 (17.702, 26.269) | -2.605 (-2.734, -2.475) | 24.824 (10.441, 39.365) | 0.748 (0.598, 0.871) | 166.954 (138.412, 195.728) | -0.381 (-0.536, -0.262) | 62.852 (55.918, 69.699) | -0.305 (-0.441, -0.185) |
| Western Sub-Saharan Africa | 22.766 (18.581, 26.956) | 2.296 (2.152, 2.429) | 48.626 (41.161, 56.193) | -0.174 (-0.245, -0.109) | 8.900 (4.600, 13.172) | 0.875 (0.809, 0.941) | 7.103 (4.844, 9.361) | -1.747 (-1.828, -1.671) | 9.467 (8.297, 10.629) | 0.371 (0.299, 0.438) |
| AAPC: average annual percentage changes; ASDR: age-standardized DALY rate; TBL: tracheal, bronchus, and lung. | | | | | | | | | | |
| *The AAPC was NOT statistically significant since its *P* value > 0.05. | | | | | | | | | | |
