# Supplementary material for: Trends, inequalities, and 2030 projections of women's tracheal, bronchial, and lung cancer attributable to air pollution, radon, and tobacco exposure
Source: Front Glob Womens Health. 2026 Aug 7;7:1662451. doi: 10.3389/fgwh.2026.1662451 (PMC13493495; doi:10.3389/fgwh.2026.1662451)
Supplement: Supplementary file 6 [file Table5.docx]

**Figure Legends**

Fig. S1 ASDR of female TBL cancer attributable to ambient particulate matter pollution (A), household air pollution from solid fuels (B), residential radon (C), smoking (D), secondhand smoke (E) in 2023, by countries.

Fig. S2 AAPC in ASDR of female TBL cancer attributable to ambient particulate matter pollution (A), household air pollution from solid fuels (B), residential radon (C), smoking (D), secondhand smoke (E) from 1990-2023, by countries.

Fig. S3 ASDR trend of female TBL cancer attributable to ambient particulate matter pollution (A), household air pollution from solid fuels (B), residential radon (C), smoking (D), secondhand smoke (E) from 1990-2023, by SDI regions.

Fig. S4 Correlations between SDIs and ASDRs of female TBL cancer attributable to ambient particulate matter pollution (A), household air pollution from solid fuels (B), residential radon (C), smoking (D), secondhand smoke (E) from 1990-2023, by GBD regions.

Fig. S5 Correlations between SDIs and ASDRs of female TBL cancer attributable to ambient particulate matter pollution (A), household air pollution from solid fuels (B), residential radon (C), smoking (D), secondhand smoke (E) in 2023, by countries. A LOESS curve was fitted to estimate the expected ASDR by SDI, and deviation was calculated as the difference between observed and predicted values. The three countries with the largest positive deviations within the low-SDI (SDI < 0.5316) and high-SDI (SDI > 0.7188) groups were labeled. Dot colors indicated whether ASDR increased or decreased between 1990 and 2023.

Fig. S6 Health concentration curve for the cumulative DALYs of female TBL cancer attributable to ambient particulate matter pollution (A), household air pollution from solid fuels (B), residential radon (C), smoking (D), secondhand smoke (E) globally, 1990-2023.

Fig. S7 Driving factors of DALY number changes in female TBL cancer attributable to ambient particulate matter pollution (A), household air pollution from solid fuels (B), residential radon (C), smoking (D), secondhand smoke (E), 1990-2023, by regions.

Fig. S8 Predicted DALYs and ASDR of female TBL cancer attributable to ambient particulate matter pollution (A), household air pollution from solid fuels (B), residential radon (C), smoking (D), secondhand smoke (E) globally, 1990-20

Fig. S1 ASDR of female TBL cancer attributable to ambient particulate matter pollution (A), household air pollution from solid fuels (B), residential radon (C), smoking (D), secondhand smoke (E) in 2023, by countries.

Fig. S2 AAPC in ASDR of female TBL cancer attributable to ambient particulate matter pollution (A), household air pollution from solid fuels (B), residential radon (C), smoking (D), secondhand smoke (E) from 1990-2023, by countries.

Fig. S3 ASDR trend of female TBL cancer attributable to ambient particulate matter pollution (A), household air pollution from solid fuels (B), residential radon (C), smoking (D), secondhand smoke (E) from 1990-2023, by SDI regions.

Fig. S4 Correlations between SDIs and ASDRs of female TBL cancer attributable to ambient particulate matter pollution (A), household air pollution from solid fuels (B), residential radon (C), smoking (D), secondhand smoke (E) from 1990-2023, by GBD regions.

Fig. S5 Correlations between SDIs and ASDRs of female TBL cancer attributable to ambient particulate matter pollution (A), household air pollution from solid fuels (B), residential radon (C), smoking (D), secondhand smoke (E) in 2023, by countries. A LOESS curve was fitted to estimate the expected ASDR by SDI, and deviation was calculated as the difference between observed and predicted values. The three countries with the largest positive deviations within the low-SDI (SDI < 0.5316) and high-SDI (SDI > 0.7188) groups were labeled. Dot colors indicated whether ASDR increased or decreased between 1990 and 2023.

Fig. S6 Health concentration curve for the cumulative DALYs of female TBL cancer attributable to ambient particulate matter pollution (A), household air pollution from solid fuels (B), residential radon (C), smoking (D), secondhand smoke (E) globally, 1990-2023.

Fig. S7 Driving factors of DALY number changes in female TBL cancer attributable to ambient particulate matter pollution (A), household air pollution from solid fuels (B), residential radon (C), smoking (D), secondhand smoke (E), 1990-2023, by regions.

Fig. S8 Predicted DALYs and ASDR of female TBL cancer attributable to ambient particulate matter pollution (A), household air pollution from solid fuels (B), residential radon (C), smoking (D), secondhand smoke (E) globally, 1990-2030.
